# A meta-learning approach for B-cell conformational epitope prediction

**DOI:** 10.1186/s12859-014-0378-y

**Published:** 2014-11-18

**Authors:** Yuh-Jyh Hu, Shun-Chien Lin, Yu-Lung Lin, Kuan-Hui Lin, Shun-Ning You

**Affiliations:** Department of Computer Science, National Chiao Tung University, 1001 University Rd., Hsinchu, Taiwan; Institute of Biomedical Engineering, National Chiao Tung University, 1001 University Rd., Hsinchu, Taiwan

**Keywords:** B-cell epitope prediction, Linear epitopes, Conformational epitopes, Meta learning

## Abstract

**Background:**

One of the major challenges in the field of vaccine design is identifying B-cell epitopes in continuously evolving viruses. Various tools have been developed to predict linear or conformational epitopes, each relying on different physicochemical properties and adopting distinct search strategies. We propose a meta-learning approach for epitope prediction based on stacked and cascade generalizations. Through meta learning, we expect a meta learner to be able integrate multiple prediction models, and outperform the single best-performing model. The objective of this study is twofold: (1) to analyze the complementary predictive strengths in different prediction tools, and (2) to introduce a generic computational model to exploit the synergy among various prediction tools. Our primary goal is not to develop any particular classifier for B-cell epitope prediction, but to advocate the feasibility of meta learning to epitope prediction. With the flexibility of meta learning, the researcher can construct various meta classification hierarchies that are applicable to epitope prediction in different protein domains.

**Results:**

We developed the hierarchical meta-learning architectures based on stacked and cascade generalizations. The bottom level of the hierarchy consisted of four conformational and four linear epitope prediction tools that served as the base learners. To perform consistent and unbiased comparisons, we tested the meta-learning method on an independent set of antigen proteins that were not used previously to train the base epitope prediction tools. In addition, we conducted correlation and ablation studies of the base learners in the meta-learning model. Low correlation among the predictions of the base learners suggested that the eight base learners had complementary predictive capabilities. The ablation analysis indicated that the eight base learners differentially interacted and contributed to the final meta model. The results of the independent test demonstrated that the meta-learning approach markedly outperformed the single best-performing epitope predictor.

**Conclusions:**

Computational B-cell epitope prediction tools exhibit several differences that affect their performances when predicting epitopic regions in protein antigens. The proposed meta-learning approach for epitope prediction combines multiple prediction tools by integrating their complementary predictive strengths. Our experimental results demonstrate the superior performance of the combined approach in comparison with single epitope predictors.

**Electronic supplementary material:**

The online version of this article (doi:10.1186/s12859-014-0378-y) contains supplementary material, which is available to authorized users.

## Background

The ability of an antibody to respond to an antigen, such as a virus capsid protein fragment, depends on the antibody’s specific recognition of an epitope, which is the antigenic site to which an antibody binds. Based on their structure and interaction with antibodies, epitopes can be divided into two categories: linear and conformational. A linear epitope is formed by a continuous sequence of amino acids, whereas a conformational epitope is composed of discontinuous primary sequences, which are close in three-dimensional space.

Several different approaches exist for predicting linear and conformational epitopes. Previous studies relied on the varying physicochemical properties of amino acids to predict linear epitopes [1–3]. A study on 484 amino acid scales revealed that predictions based on the best-performing scales poorly correlated with experimentally confirmed epitopes [4]. This result prompted the development of machine-learning methods to improve prediction. BepiPred combines amino acid propensity scales with a hidden Markov model to achieve marginal improvement over methods based on physicochemical properties [5]. ABCPred uses artificial neural networks (ANN) for predicting linear B-cell epitopes [6]. Chen et al. proposed the novel amino acid pair (AAP) antigenicity scale [7], for which the authors trained a support vector machine (SVM) classifier, using the AAP propensity scale to distinguish epitopes and nonepitopes. BCPREDS uses SVM combined with a variety of kernel methods, including string kernels, radial basis kernels, and subsequence kernels, to predict linear B-cell epitopes [8].

An increase in the availability of protein structures has enabled the identification of conformational epitopes by using various computational methods. For example, DiscoTope 2.0 uses a combination of amino acid composition information, spatial neighborhood information, and a surface measure for predicting epitopes [9]. ElliPro uses Thornton’s propensities and applies residue clustering to identify epitopes [10]. SEPPA 2.0 predicts conformational epitopes based on the unit patches of residue triangles, and the clustering coefficient for describing local spatial context and compactness with two new parameters appended, ASA (Accessible Surface Area) propensity, and consolidated amino acid index [11]. EPITOPIA combines structural and physiochemical features, and adopts a Bayesian classifier to predict epitopes [12]. EPSVR uses a support vector regression method to predict conformational epitopes. The meta learner EPMeta incorporates consensus results from multiple prediction servers by using a voting mechanism [13].

In this study, we propose combining multiple predictions to improve epitope prediction based on two meta-learning strategies: stacked generalization (stacking) [14,15] and cascade generalization (cascade) [16,17]. These strategies work in a hierarchical architecture of meta learners and base learners, in which the input space for meta learners is extended by the predictions of the base learners. We selected several linear and conformational epitope predictors as the base learners, and evaluated four inductive learning algorithms as the meta learners. To evaluate performance, we tested the combinatorial method on an independent set of antigen proteins that were not used previously to train the epitope prediction tools according to the documents on the tools and their publications. Our results indicate the potential of meta learning for epitope prediction.

## Results and discussion

### Prediction correlations between base learners

For a meta-learning method to perform effectively, the base learners must have complementary predictive capabilities, which can be reflected by relatively low correlation among their predictions. We selected four conformational and four linear epitope predictors as our base learners. The conformational predictors were DiscoTope 2.0 [9], ElliPro [10], SEPPA 2.0 [11], and Bpredictor [18], and the linear epitope predictors were BepiPred [5], ABCpred [6], AAP [7], and BCPREDS [8]. We calculated the Pearson’s correlation coefficients for the prediction scores produced by the base prediction tools. To further analyze the correlations among predictions based on the score rankings, we sorted the prediction scores of all protein residues provided by each base learner and then conducted a Spearman’s rank correlation analysis. Tables 1 and 2 list the Pearson’s correlation coefficients and Spearman's rank correlation coefficients of all pairs of linear and conformational predictors, respectively. The average correlation coefficients of the linear and conformational prediction tools were 0.383 vs. 0.384 and 0.370 vs. 0.459 in the Pearson’s and Spearman’s correlation analyses, respectively, which indicate a relatively weak correlation among the epitope predictions of the base learners.

**Table 1:** **Correlation analysis of linear epitope predictors**

**Linear**	**AAP**		**ABCpred**		**BCPREDS**	
	**Pearson**	**Spearman**	**Pearson**	**Spearman**	**Pearson**	**Spearman**
AAP	1	1	-	-	-	-
ABCpred	0.241	0.251	1	1	-	-
BCPREDS	0.515	0.520	0.342	0.287	1	1
BepiPred	0.383	0.372	0.282	0.299	0.536	0.489

**Table 2:** **Correlation analysis of conformational epitope predictors**

**Conformational**	**SEPPA 2.0**		**DiscoTope 2.0**		**Bpredictor**	
	**Pearson**	**Spearman**	**Pearson**	**Spearman**	**Pearson**	**Spearman**
SEPPA 2.0	1	1	-	-	-	-
DiscoTope 2.0	0.246	0.400	1	1	-	-
Bpredictor	0.339	0.509	0.372	0.364	1	1
ElliPro	0.333	0.487	0.388	0.362	0.624	0.630

**Figure 1:**
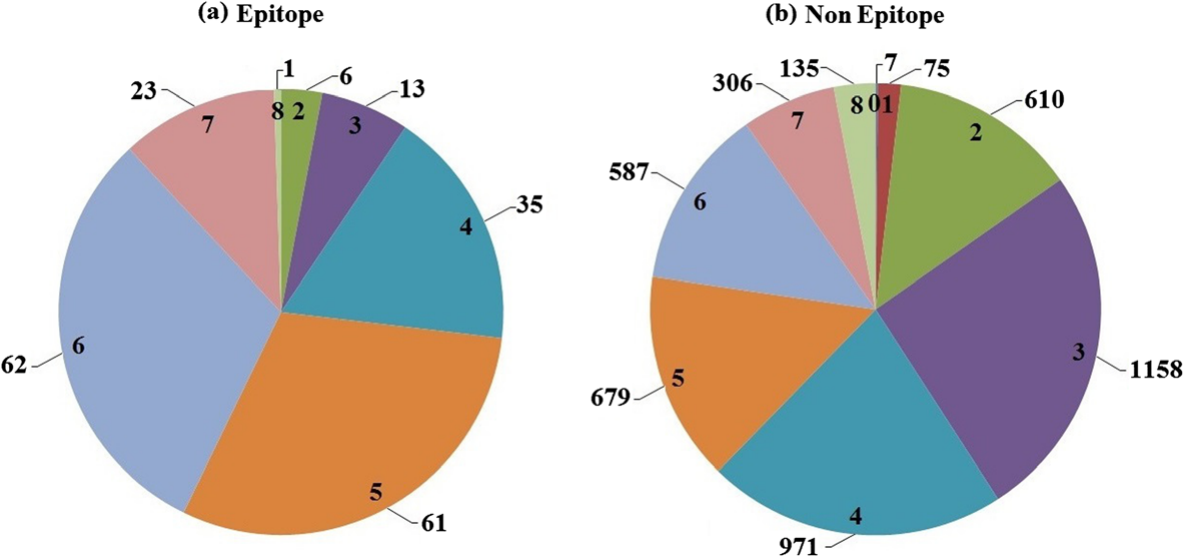
**Pie charts showing the degree of agreement among the base tools for epitope prediction. (a)** Distribution of the counts of epitope residues based on the number of base tools with the same prediction, and **(b)** distribution of the counts of nonepitope residues based on the number of base tools with the same classification.

In the independent test data set, 201 epitope residues and 4528 nonepitope residues exist on 15 protein antigens. A base predictor can classify a protein residue as epitopic or nonepitopic. For each of the 201 epitope residues, we counted the number of base tools that correctly classified the residue as epitopic. Similarly, for each of the 4528 nonepitope residues, we counted the number of base tools that correctly classified the residue as nonepitopic. Figure 1 shows the distributions of epitope and nonepitope residues for the independent test proteins, based on the number of base tools with the same prediction, to indicate the degree of agreement in classification among the base tools. For example, in Figure 1(a), we observed 6 epitope residues, each of which was classified correctly by two of the base predictors. Overall, we observed that none of the epitope residues were classified correctly by only one of the base tools, or escaped the detection of all of the base predictors, and 11% and 0.5% of the epitope residues were classified correctly by seven and all of the base tools, respectively. By contrast, >85% of the epitope residues were classified correctly by between three and six base predictors. We observed similar trends for the nonepitope residues. These results indicate that base learners do not always agree when predicting epitopes, and may have complementary strengths, suggesting that a meta learner built upon these learners can demonstrate synergy in their predictive capabilities.

### Performances of meta classifiers and base learners

The multilevel architecture for stacked or cascade generalization can vary with the arrangement of the meta learners in the hierarchy. For example, we can place SVM [19] at the top level in a stacked generalization architecture, or we can substitute C4.5 [20] for SVM. For cascade generalization, we can place the k-Nearest-Neighbor (k-NN) [21] prior to the ANN [22], or vice versa, in the cascading sequence. We conducted two stratified five-fold cross-validations (CV) to evaluate the performances of different architectures. We randomly divided a data set of 94 antigens into five disjoint folds (i.e., subsets), each of approximately equal size. We stratified the folds to maintain the same distribution of epitopes and nonepitopes as in the original data set. We used one fold of data for testing prediction performance, and used the remaining four folds for training. We repeated the same training–testing process on each fold iteratively. Each run produced a result based on the fold selected for testing. The overall performance was used as the average of the results obtained from all iterations of the two 5-fold CVs.

**Figure 2:**
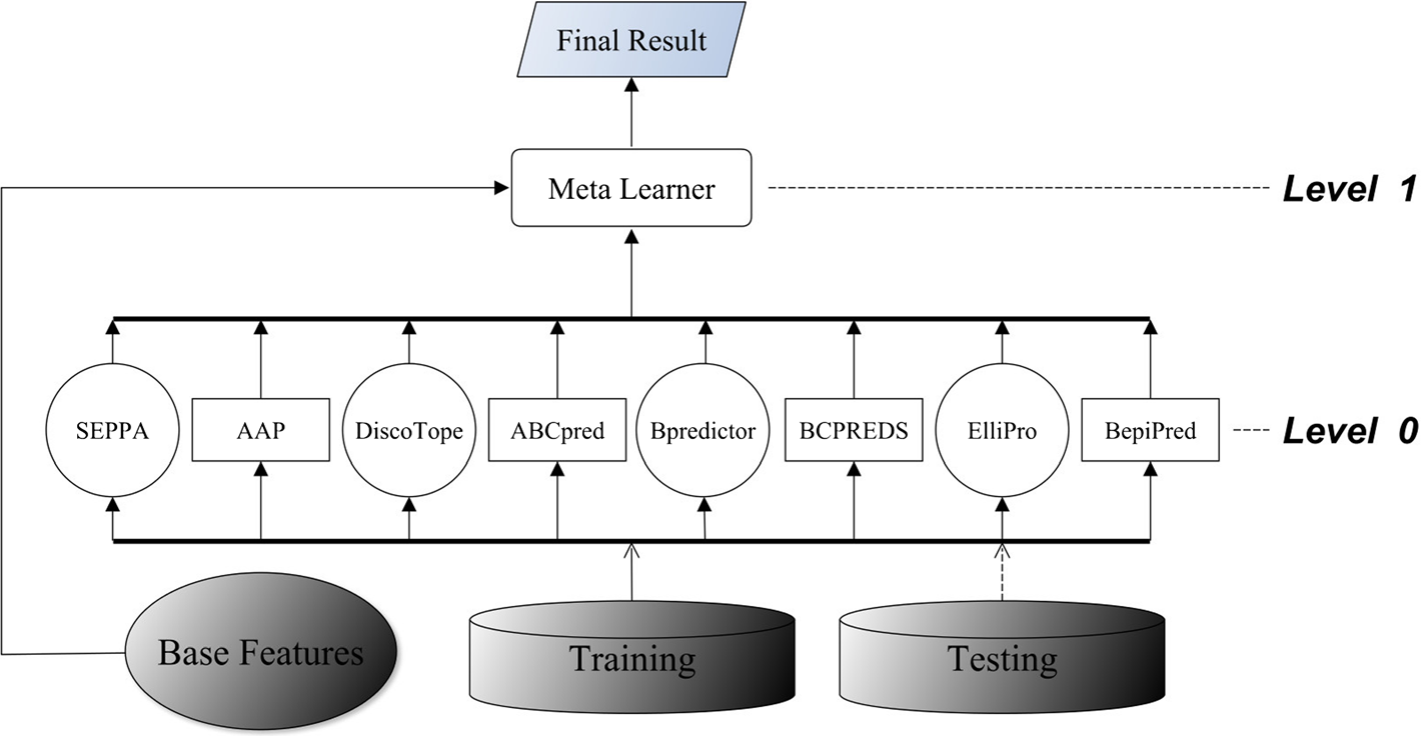
**Two-level stacking architecture.** The conformational epitope predictors and linear epitope predictors were all placed at Level 0. One of the learners SVM, C4.5, k-NN, or ANN served as a meta learner to integrate the output from the base predictors, and produced the meta classification as the final result.

**Figure 3:**
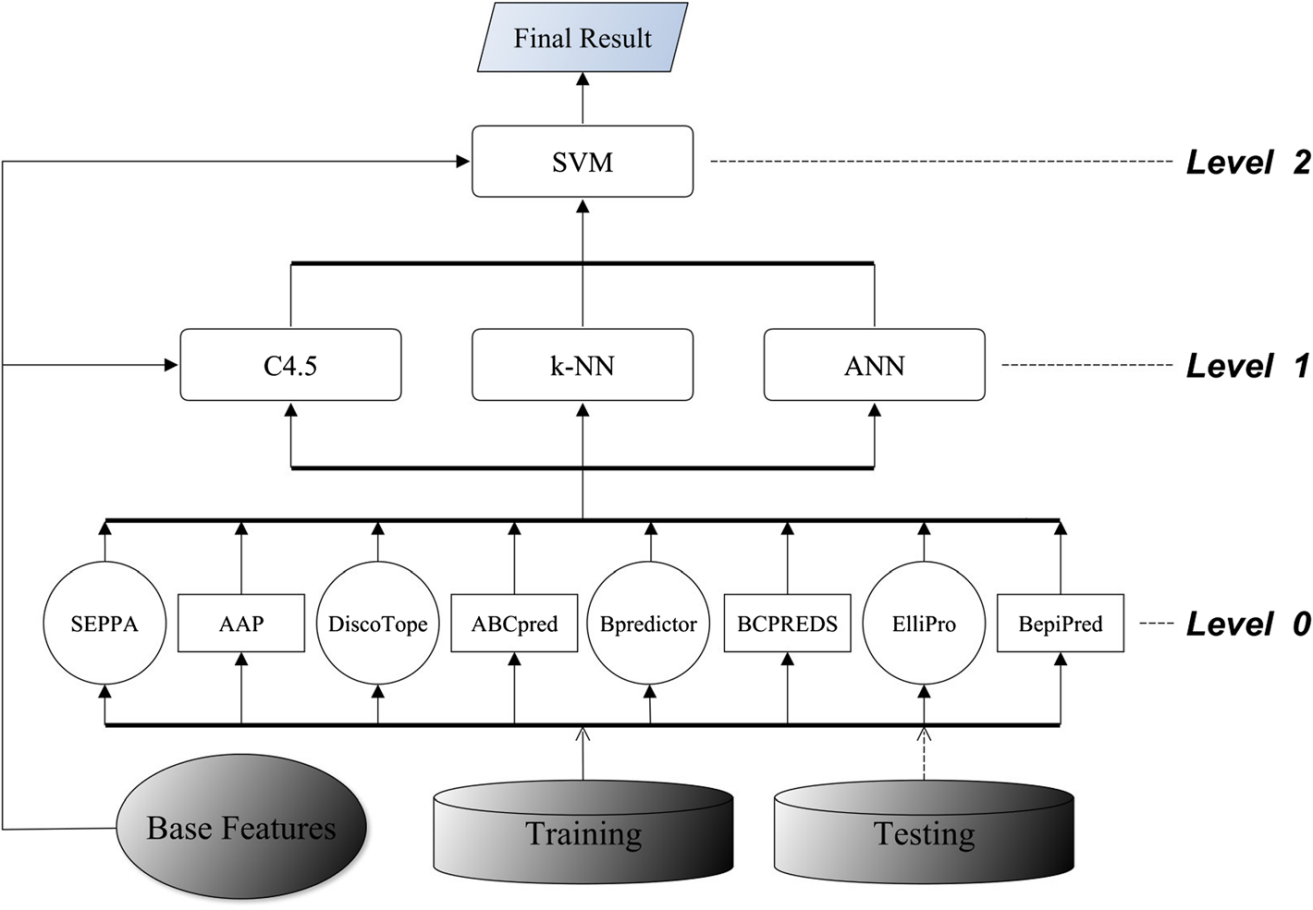
**Three-level stacking architecture.** The conformational epitope predictors and linear epitope predictors were all placed at Level 0. We selected C4.5, k-NN, and ANN as the Level-1 meta learners that transformed the output of the base predictors into meta features, and passed them to the successive level. We designated SVM as the top meta learner that learned from the base features and the meta features to produce the meta classification as the final result.

**Figure 4:**
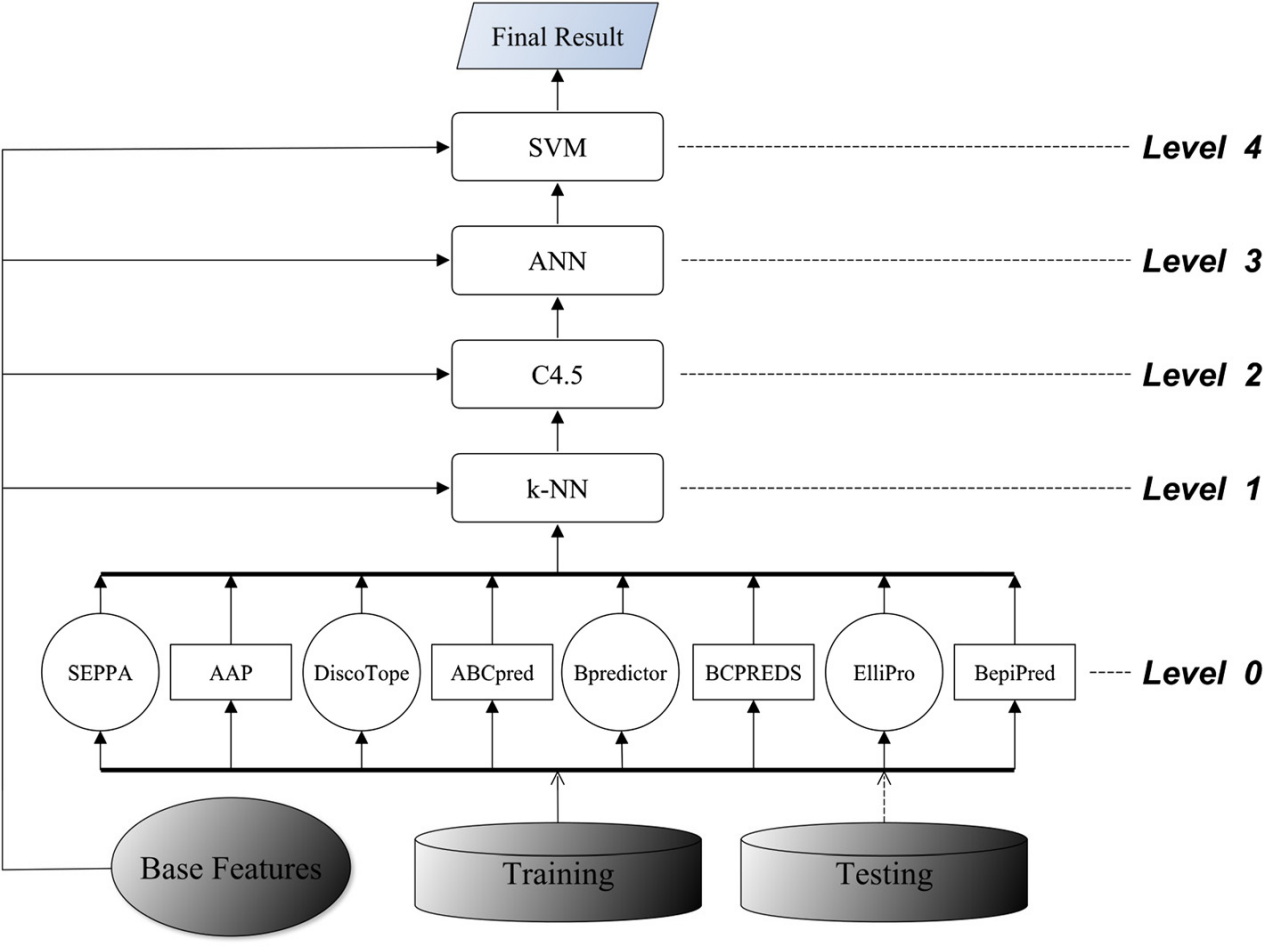
**Cascade generalization architecture.** The conformational epitope predictors and linear epitope predictors all served at Level 0 as the base predictors. We placed k-NN, C4.5, ANN, and SVM sequentially from Levels 1 to 4 as meta learners. Each meta learner generalized the output from the previous level to meta knowledge in the form of meta features. The meta features and base features propagated sequentially to the successive level as input to the subsequent meta learner. The top-level meta learner, SVM, produced the final meta classification.

First, we tested C4.5, k-NN (k =3), ANN, and SVM in a two-level (Levels 0 and 1) stacking architecture (Figure 2). Table 3 shows the average performances of the two 5-fold CVs. The results indicated that SVM was the best-performing meta learner when compared with C4.5, k-NN, and ANN. Therefore, to build a three-level (Levels 0–2) stacked generalization architecture we placed SVM above the other three classifiers to arbitrate their predictions. Figure 3 shows the three-level stacked architecture. Cascade generalization performs a sequential composition of meta learners in a hierarchy in which only one meta learner exists at each level. We tested all 24 possible sequential arrangements of the meta learners SVM, C4.5, k-NN, and ANN by using CV. Figure 4 shows the best-performing cascade generalization architecture.

**Table 3:** **Five-fold cross-validations of meta classifiers**

**Classifier**	**TPR**	**FPR**	**Precision**	**Accuracy**	**F-score**	**MCC**	**AUC**
2-level (ANN)^a^	0.514	0.019	0.705	0.944	0.594	0.573	0.748
2-level (C4.5)^a^	0.511	0.023	0.663	0.941	0.577	0.551	0.744
2-level (k-NN)^a^	0.496	0.012	0.783	0.949	0.607	0.599	0.742
2-level (SVM)^a^	0.593	0.009	0.848	0.959	0.697	0.689^d^	0.920
3-level Stacking^b^	0.579	0.009	0.850	0.958	0.689	0.682^d^	0.925
Cascade^c^	0.588	0.010	0.843	0.959	0.693	0.684^d^	0.925

^a^Two-level stacking meta classifiers with ANN, C4.5, k-NN, or SVM as the top-level meta learner.

^b^Three-level stacking meta classifier (Figure 3).

^c^Cascade meta classifier (Figure 4).

^d^Paired *t* test showed no significant difference.

Table 3 shows the average results of the two 5-fold CVs for the three-level stacked and cascade generalizations. Table 4 presents the performances of each base learner based on the same CV. We optimized all of the parameters of the base predictors or meta learners by using a systematic search (a sequential or grid search [23]) within a range of parameter values in the CVs. We selected the optimum parameter values and used them in subsequent independent tests and ablation studies. Table 5 lists the parameter values for the base epitope predictors. Tables 3 and 4 show that stacking and cascade markedly outperformed all of the base prediction tools for accuracy, F-score, Matthews correlation coefficient (MCC), and area under the curve (AUC). The differences among the meta-learning models were nonsignificant in a paired *t* test. These results demonstrate the advantages of exploiting the complementary capabilities of the base prediction tools.

**Table 4:** **Five-fold cross-validations of base epitope predictors**

**Classifier**	**TPR**	**FPR**	**Precision**	**Accuracy**	**F-score**	**MCC**	**AUC**
SEPPA 2.0	0.450	0.097	0.291	0.867	0.348	0.290	0.793
DiscoTope 2.0	0.930	0.761	0.096	0.294	0.173	0.110	0.617
Bpredictor	0.129	0.017	0.399	0.916	0.195	0.192	0.690
ElliPro	0.711	0.512	0.108	0.506	0.186	0.109	0.635
AAP	0.831	0.770	0.085	0.278	0.154	0.039	0.490
ABCpred	0.603	0.548	0.088	0.463	0.152	0.031	0.536
BCPREDS	0.962	0.906	0.084	0.163	0.154	0.053	0.476
BepiPred	0.718	0.500	0.110	0.517	0.191	0.118	0.609

**Table 5:** **Parameter settings in base epitope predictors**

**Base learner**	**Parameter**	**Range of parameter values**	**Selected value**
SEPPA 2.0	scoring threshold	0.00 ~ 1.00	0.21
DiscoTope 2.0	scoring threshold	−70.00 ~ 10.00	−18.09
Bpredictor	scoring threshold	0.00 ~ 1.00	0.88
ElliPro	scoring threshold	0.00 ~ 1.00	0.44
AAP	window size	10, 12, 14, 16, 18, 20	16
ABCpred	scoring threshold	0.00 ~ 1.00	0.84
BCPREDS	window size	12, 14, 16, 18, 20, 22	20
BepiPred	scoring threshold	−4.00 ~ 3.00	0.02

**Figure 5:**
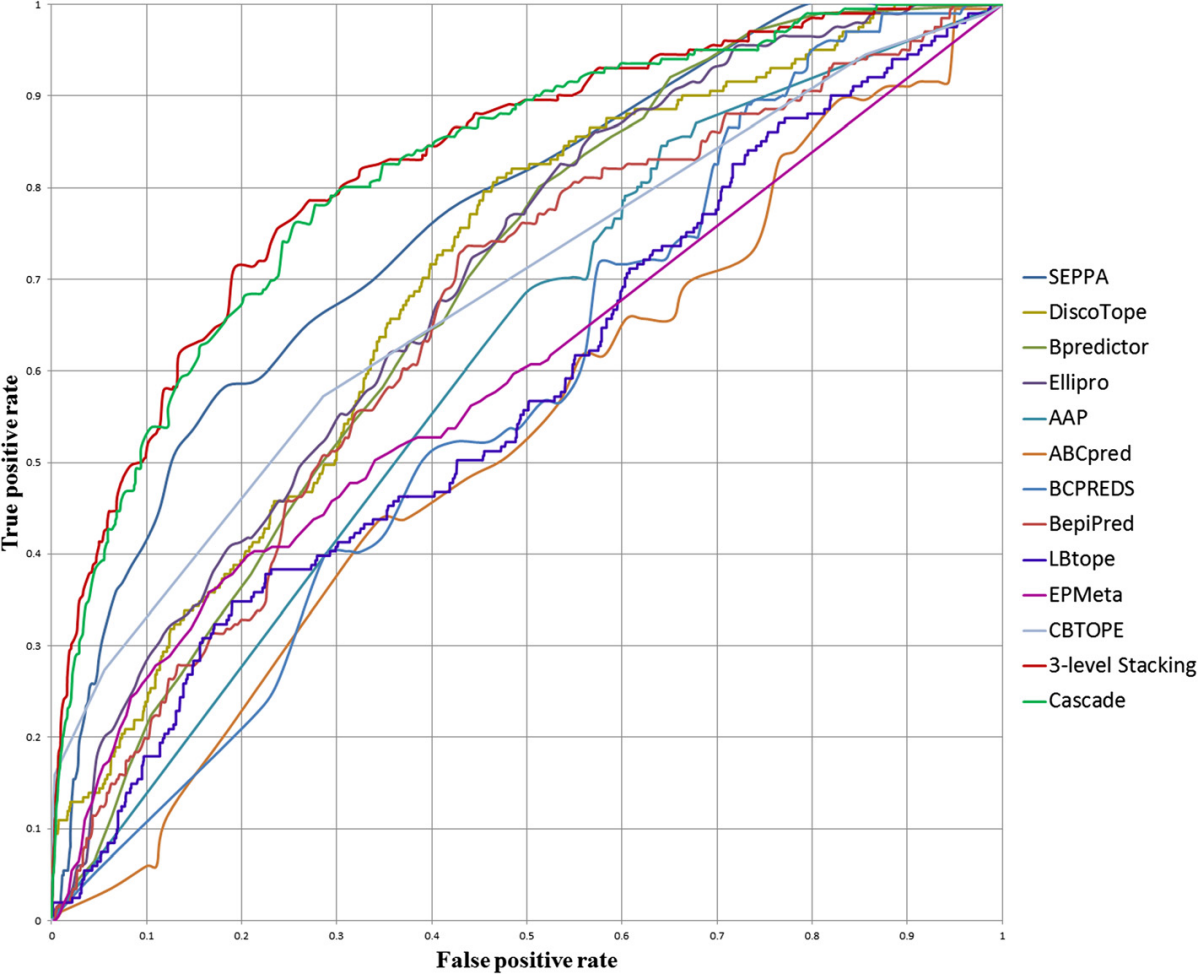
**The ROC curves of epitope predictors and meta classifiers based on the independent test data.** The curves show the amounts of TP and FP produced by the classifiers in different parameter settings. The results show that both stacking and cascade outperformed all other epitope prediction tools, including the meta server, EPMeta, in the independent test.

Subsequently, we conducted an independent test using the test data set of 15 antigens that were not used previously to train the base learners, and a training data set of 94 antigens that were known from the literature or the websites to train the base learners. To ensure a fair comparison, we used the training data to train a meta classifier, and compared its performance with the performances of the base learners for the same test data. This independent test provided consistent and unbiased comparisons among the proposed meta-learning approach and the eight base learners. We also included two recent epitope prediction methods, CBTOPE [24], LBtope [25], for comparison. We selected the parameter values of their best-performing models for the training data set, respectively, and used them in the independent test to ensure a fair comparison. Table 6 shows the results, which indicate marked differences in accuracy, F-score, MCC, and AUC among the meta models and the base learners. Figure 5 shows the ROC curves. The ROC curves indicate the trade-off between the amounts of true positives (TP) and false positives (FP) produced by the classifiers.

**Table 6:** **Results of the independent test data**

**Classifier**	**TPR**	**FPR**	**Precision**	**Accuracy**	**F-score**	**MCC**	**AUC**
SEPPA 2.0	0.289	0.050	0.204	0.922	0.239	0.202	0.765
DiscoTope 2.0	0.930	0.763	0.051	0.266	0.097	0.080	0.699
Bpredictor	0.010	0.007	0.057	0.951	0.017	0.006	0.683
ElliPro	0.826	0.535	0.064	0.480	0.119	0.118	0.696
AAP	0.846	0.641	0.055	0.379	0.104	0.086	0.609
ABCpred	0.507	0.480	0.045	0.519	0.082	0.011	0.530
BCPREDS	0.990	0.874	0.048	0.163	0.091	0.072	0.570
BepiPred	0.761	0.499	0.063	0.512	0.117	0.106	0.656
CBTOPE^a^	0.159	0.003	0.681	0.961	0.258	0.317	0.681
LBtope^b^	0.632	0.578	0.046	0.431	0.086	0.022	0.575
EPMeta^c^	0.129	0.043	0.118	0.922	0.124	0.083	0.595
3-level Stacking	0.194	0.008	0.520	0.958	0.283	0.300	0.793
Cascade	0.199	0.008	0.519	0.958	0.288	0.304	0.789

^a^We selected the parameter value (0.7) of the best-performing CBTOPE on the training data set of 94 antigens, and used the value in the independent test. CBTOPE’s performances were markedly lower for ACC, F-score, and MCC (0.708, 0.143, and 0.126), using the default value (−0.3).

^b^We selected the parameter value (42.4) of the best-performing LBtope on the training data set of 94 antigens, and used the value in the independent test. By contrast, LBtope’s performances were markedly higher for ACC, F-score, and MCC (0.803, 0.123, and 0.077), using the default value (60).

^c^We selected the parameter value (86) of the best-performing EPMeta on the training data set of 94 antigens, and used the value in the independent test. EPMeta did not provide the default parameter value.

From the results of the 5-fold CVs and the independent test, we observed that most of the base tools produced high true positive rates of prediction, nevertheless they also suffered high false positive rates. In contrast to most of the base tools, the proposed meta-learning approach (stacking and cascade) showed lower false positive rates. Although Bpredictor demonstrated the lowest false positive rate in the independent test, unfortunately its true positive rate was also the lowest. Among the eight base prediction tools, SEPPA 2.0 obtained the best balance between true and false positive rates as indicated by the highest F-score, MCC, and AUC. When comparing SEPPA 2.0 with stacking and cascade, we observed that both stacking and cascade outperformed SEPPA 2.0 for all the performance measures except the true positive rate in the independent test. Overall, these observations suggest that the performance of an ensemble approach based on meta learning is superior to that of a single prediction tool for B-cell epitope prediction.

The applicability of an epitope predictor is limited by the protein properties it explores, and constrained by the search strategies it adopts. One method for achieving improvement is combining the results from an ensemble of various predictors. Different ensemble approaches are distinguished by the manner in which they integrate the results from a set of predictors with different characteristics. We compared the proposed meta-learning approach with a meta server, EPMeta, which incorporates the consensus results from multiple discontinuous epitope predictors by using a multistage voting scheme [13]. Although EPMeta and the other base learners performed comparably, both the proposed stacking and cascade meta classifiers markedly outperformed EPMeta for F-score, MCC, and AUC.

In addition to the comparison between the meta classifiers and the base epitope predictors for the same independent test data set of 15 antigens, we also compared the meta classifiers with the epitope predictors separately, using different data sets. We conducted the experiments on several representative epitope predictors released in 2008–2014: SEPPA 2.0 (2014), DiscoTope 2.0 (2012), Bpredictor (2011), CBTOPE (2010), and ElliPro (2008). Each of them had been trained and tested by different data sets [9–11,18,24]. In each experiment, we selected one epitope predictor for comparison. If it was one of the eight base learners, we built the meta classifier upon the remaining seven base learners. We only trained and tested the meta classifier on the same data sets that had been used specifically to train and test the predictor selected for comparison. Other predictors, such as ABCpred, BCPREDS, and LBtope, though their data sets were available, were excluded from the experiments because their data sets either contained only sequence segments, or lacked the information of PDB files required to calculate the structure-based feature values, such as ASA, for the meta classifiers. Table 7 shows that stacking and cascade outperformed SEPPA 2.0, DiscoTope 2.0, Bpredictor, CBTOPE, and ElliPro markedly in the individual tests. The results demonstrate that the synergy in the effects of multiple epitope predictors can achieve superior performance compared with that produced by a single epitope predictor.

**Table 7:** **Results of individual comparisons**

**Classifier**	**TPR**	**FPR**	**Precision**	**Accuracy**	**F-score**	**MCC**	**AUC**
SEPPA 2.0	0.155	0.045	0.161	0.913	0.158	0.112	0.697
3-level Stacking^a^ w/o SEPPA 2.0	0.418	0.030	0.384	0.935	0.386	0.351	0.820
Cascade^a^ w/o SEPPA 2.0	0.404	0.033	0.405	0.937	0.404	0.371	0.820
DiscoTope 2.0	0.917	0.625	0.090	0.409	0.164	0.148	0.748
3-level Stacking^a^ w/o DiscoTop 2.0	0.231	0.013	0.541	0.939	0.324	0.327	0.809
Cascade^a^ w/o DiscoTope 2.0	0.212	0.013	0.532	0.939	0.303	0.310	0.806
Bpredictor	0.045	0.028	0.067	0.933	0.054	0.021	0.683
3-level Stacking^b^ w/o Bpredictor	0.119	0.006	0.471	0.957	0.190	0.222	0.779
Cascade^b^ w/o Bpredictor	0.149	0.002	0.769	0.962	0.250	0.328	0.787
ElliPro	0.421	0.279	0.131	0.694	0.199	0.090	0.630
3-level Stacking^c^ w/o ElliPro	0.367	0.009	0.802	0.935	0.504	0.516	0.861
Cascade^c^ w/o ElliPro	0.346	0.010	0.770	0.932	0.478	0.488	0.857
CBTOPE^d^	0.801	0.424	0.118	0.591	0.205	0.188	0.798
3-level Stacking^d^	0.446	0.010	0.751	0.954	0.558	0.557	0.913
Cascade^d^	0.446	0.010	0.762	0.954	0.562	0.562	0.908

^a^Meta classifiers were trained and tested using the data sets that were used specifically to train and test SEPPA 2.0 (or DiscoTope 2.0), excluding the antigens with missing feature values. All the classifiers, including the base predictor (SEPPA 2.0 or DiscoTope 2.0), were tested on the same test data to conduct a consistent comparison.

^b^Meta classifiers were trained on the data used specifically to train Bpredictor, excluding the antigens with missing feature values. Though Bpredictor provided the test data set of its own, the data lacked the epitope residues annotated in the IEDB. Alternatively, we used the independent test data set of 15 antigens (Table 15) to test all the classifiers, including Bpredictor, to conduct a consistent comparison.

^c^ElliPro only provided the test data set, but no training data. Meta classifiers were consequently trained on the training data set of 94 antigens (Table 16), and tested on the test data of ElliPro, excluding the antigens with missing feature values. All the classifiers, including ElliPro, were tested on the same test data to conduct a consistent comparison.

^d^Meta classifiers were trained and tested using the non-redundant (<40% sequence identity) benchmark dataset previously used to evaluate CBTOPE, excluding the antigens with missing feature values. Following CBTOPE, we adopted 5-fold CV to compare the performances. All the classifiers, including CBTOPE, were tested on the same test data to conduct a consistent comparison. The parameter value (−0.3) we used for CBTOPE was the same as used previously to evaluate CBTOPE in [24].

### Ablation analysis

In a meta-learning architecture, base learners interact and contribute differently to the meta decision. An ablation study provides insight into the effects of base learners on the prediction performance of a meta classifier. However, the time required for a complete ablation analysis increases exponentially with the number of base learners. To avoid computational explosion, we adopted a greedy approach for the ablation study. Although the greedy ablation analysis does not consider all possible combinations of base learners in a meta-learning architecture, it provides a significant estimate for the base learners in meta classification.

We used conformational (DiscoTope 2.0, ElliPro, SEPPA 2.0, and Bpredictor) and linear (BepiPred, ABCpred, AAP, and BCPREDS) epitope predictors as the base learners for our meta classifiers. We conducted three ablation analyses for the stacking and cascade meta classifiers. The first analysis investigated a tool-based meta classifier and a feature-based meta classifier. The tool-based meta classifier only used the eight epitope prediction tools as the base learners in the meta learning hierarchy, whereas the feature-based meta classifier only adopted the structure-related features, such as secondary structures [26], and the features that were relevant to the protein sequences, such as amino acid hydrophilicity [27]. Tables 8 and 9 show the performances of the two types of meta classifier. The results indicated a marked reduction in prediction performance for F-score, MCC, and AUC when the meta classifier used only the base tools, or only the base features. Prior to ablation, the performances of the stacking meta classifier for F-score, MCC, and AUC were 0.283, 0.300, and 0.793, respectively. After ablation, they reduced to 0.103, 0.133, and 0.663, respectively, for the tool-based stacking meta classifier, and to 0.109, 0.134, and 0.658, respectively, for the feature-based stacking meta classifier. We observed similar trends for the cascade meta classifier. Collectively, the base predictors and features exerted great influence on the meta classification, as suggested by the result that the removal of the predictors or the features induced a substantial reduction in prediction performance. In addition, we observed approximately equal amount of reduction in performance after the removal of the base tools or the base features, which indicated the comparable influence of the predictors and the features on the learned meta model. Extending the input space for the meta learners with both the predictions of the base tools and the protein features improved the prediction of epitopes (Tables 6, 8, and 9). Overall, these results demonstrate synergy in the effects of the conformational and linear epitope predictors, and protein features in meta learning.

**Table 8:** **Ablation analysis of 3-level stacking meta classifiers**

**Classifier**	**TPR**	**FPR**	**Precision**	**Accuracy**	**F-score**	**MCC**	**AUC**
Tool-based	0.060	0.005	0.364	0.956	0.103	0.133	0.663
Feature-based	0.065	0.006	0.342	0.955	0.109	0.134	0.658

**Table 9:** **Ablation analysis of cascade meta classifiers**

**Classifier**	**TPR**	**FPR**	**Precision**	**Accuracy**	**F-score**	**MCC**	**AUC**
Tool-based	0.060	0.008	0.245	0.952	0.096	0.103	0.648
Feature-based	0.060	0.007	0.279	0.953	0.098	0.112	0.648

The second analysis focused on the interactions of the eight epitope predictors, and their individual influence on the meta decision. We applied a greedy iterative backward elimination approach for ablation analysis to avoid exponential computational time, using MCC as the performance measure. We adopted MCC in the ablation study because it considers all four numbers (TP, FP, TN, and FN), and provides a more balanced evaluation of prediction than some measures, such as TPR or precision [28]. In each iteration, we removed the base learner from the meta classifier if its removal caused a maximal decrease in MCC. Table 10 shows the order of the base learners removed sequentially from the stacking meta classifier. SEPPA 2.0, ElliPro, and BepiPred were the first three base tools to be removed from the stacking meta classifier. This observation agreed with our expectation that their removal would induce a maximal reduction in performance because SEPPA 2.0, ElliPro, and BepiPred were the top three base predictors in the independent test according to MCC (Table 6). The rest of the order varied because of the removal of the base tools. The remaining two conformational predictors, Bpredictor and DiscoTope 2.0, were the sixth and seventh to be removed after the selection of BCPREDS and AAP for removal. The final base tool to be removed was the linear epitope predictor ABCpred.

**Table 10:** **Ablation analysis of base learner interactions in stacking meta classifiers**

**Classifier** ^*^	**TPR**	**FPR**	**Precision**	**Accuracy**	**F-score**	**MCC**	**AUC**
3-level stacking	0.194	0.008	0.520	0.958	0.283	0.300	0.793
\SEPPA 2.0	0.169	0.009	0.447	0.956	0.245	0.256	0.755
\ElliPro	0.144	0.012	0.349	0.952	0.204	0.203	0.746
\BepiPred	0.144	0.012	0.341	0.952	0.203	0.200	0.749
\BCPREDS	0.109	0.009	0.338	0.953	0.165	0.173	0.717
\AAP	0.124	0.016	0.255	0.947	0.167	0.153	0.758
\Bpredictor	0.154	0.012	0.356	0.952	0.215	0.213	0.724
\DiscoTope 2.0	0.045	0.006	0.257	0.954	0.076	0.092	0.672
\ABCpred	0.065	0.006	0.342	0.955	0.109	0.134	0.658

^*^Classifiers tested in the ablation analysis. The first classifier in the first row is the stacking meta classifier that employs all of the 8 base learners (Figure 3). The remaining classifiers are listed in the order in which they were selected to be removed iteratively from the stacking meta classifier for the ablation study. ‘\’ indicates “removed”. For example, the second classifier is the stacking meta classifier after SEPPA 2.0 was removed, and the third classifier is the stacking meta classifier after SEPPA 2.0 and ElliPro were removed from the meta model. The meta classifier in the final row did not apply any base learner after the final prediction tool ABCpred was removed.

In contrast to the results of the stacking meta classifier, although the linear epitope predictor AAP did not perform the best for MCC in the independent test, it was the first to be removed from the cascade meta classifier (Table 11). SEPPA 2.0 outperformed the other base predictors in the independent test, however it was the fourth to be removed after AAP, ElliPro and Bpredictor had been selected for removal. Prior to the remaining conformational prediction tool DiscoTope 2.0, we removed the linear epitope predictor BCPREDS, and the rest of the linear epitope predictors, BepiPred and ABCpred, were the last two base tools to be removed.

**Table 11:** **Ablation analysis of base learner interactions in cascade meta classifiers**

**Classifier** ^*^	**TPR**	**FPR**	**Precision**	**Accuracy**	**F-score**	**MCC**	**AUC**
Cascade	0.199	0.008	0.519	0.958	0.288	0.304	0.789
\AAP	0.164	0.010	0.423	0.955	0.237	0.244	0.774
\ElliPro	0.144	0.009	0.414	0.955	0.214	0.226	0.748
\Bpredictor	0.149	0.009	0.435	0.956	0.222	0.237	0.743
\SEPPA 2.0	0.065	0.003	0.500	0.957	0.115	0.169	0.698
\BCPREDS	0.025	0.005	0.179	0.954	0.044	0.052	0.695
\DiscoTope 2.0	0.045	0.004	0.321	0.955	0.079	0.107	0.684
\BepiPred	0.065	0.006	0.317	0.954	0.107	0.127	0.653
\ABCpred	0.060	0.007	0.279	0.953	0.098	0.112	0.648

^*^Classifiers tested in the ablation analysis. The first classifier in the first row is the cascade meta classifier that employs all of the 8 base learners (Figure 4). The remaining classifiers are listed in the order in which they were selected to be removed iteratively from the cascade meta classifier for the ablation study. ‘\’ indicates “removed”. For example, the second classifier is the cascade meta classifier after AAP was removed, and the third classifier is the cascade meta classifier after AAP and ElliPro were removed from the meta model. The meta classifier in the final row did not apply any base learner after the final prediction tool ABCpred was removed.

The third ablation analysis evaluated the individual influence of the linear base prediction tools on the meta classifiers. We applied a greedy iterative forward selection approach starting with a meta classifier built upon all the conformational base learners. In each iteration, we added one linear base learner if its addition caused a maximal increase in MCC. Tables 12 and 13 show the order of the linear tools added sequentially to the stacking and the cascade meta classifiers, respectively. In both analyses, BCPREDS was the first selected linear base tool added to the meta classifiers. Though it was not the top linear tool in the 5-fold CVs and the independent test according to MCC, it had the highest TPR compared with the other linear tools. The addition of BCPREDS to the conformational meta classifiers increased the TPRs markedly for stacking and cascade, from 0.144 and 0.154 to 0.184 and 0.204, respectively. In addition, after the inclusion of BCPREDS, both stacking and cascade achieved their maximal MCCs, 0.317 and 0.327, respectively. Though the rest of the order varied between stacking and cascade because of the addition of other linear base learners, the performances of the meta classifiers for MCC remained above (or equal to) 0.3.

**Table 12:** **Ablation analysis of influence of linear base learners on stacking meta classifiers**

**Classifier** ^*^	**TPR**	**FPR**	**Precision**	**Accuracy**	**F-score**	**MCC**	**AUC**
Conformational 3-level Stacking	0.144	0.018	0.261	0.946	0.186	0.168	0.801
+BCPREDS	0.184	0.006	0.597	0.960	0.281	0.317	0.753
+AAP	0.194	0.008	0.527	0.958	0.284	0.303	0.788
+ BepiPred	0.214	0.009	0.524	0.958	0.304	0.317	0.788
+ABCpred	0.194	0.008	0.520	0.958	0.283	0.300	0.793

^*^Classifiers tested in the ablation analysis. The first classifier in the first row is the stacking meta classifier that only employs the 4 conformational base learners. The remaining classifiers are listed in the order in which they were selected to be added iteratively to the stacking meta classifier for the ablation study. ‘+’ indicates “added.” For example, the second classifier is the stacking meta classifier after BCPREDS was added, and the third classifier is the stacking meta classifier after BCPREDS and AAP were added to the meta model. The meta classifier in the final row applied all the base learners after the final prediction tool ABCpred was added.

**Table 13:** **Ablation analysis of influence of linear base learners on cascade meta classifiers**

**Classifier** ^*^	**TPR**	**FPR**	**Precision**	**Accuracy**	**F-score**	**MCC**	**AUC**
Conformational cascade	0.154	0.010	0.397	0.954	0.222	0.228	0.743
+BCPREDS	0.204	0.007	0.577	0.960	0.301	0.327	0.745
+ABCpred	0.184	0.005	0.617	0.960	0.284	0.323	0.760
+AAP	0.189	0.006	0.603	0.960	0.288	0.323	0.765
+ BepiPred	0.199	0.008	0.519	0.958	0.288	0.304	0.789

^*^Classifiers tested in the ablation analysis. The first classifier in the first row is the cascade meta classifier that only employs the 4 conformational base learners. The remaining classifiers are listed in the order in which they were selected to be added iteratively to the cascade meta classifier for the ablation study. ‘+’ indicates “added”. For example, the second classifier is the cascade meta classifier after BCPREDS was added, and the third classifier is the cascade meta classifier after BCPREDS and ABCpred were added to the meta model. The meta classifier in the final row applied all the base learners after the final prediction tool BepiPred was added.

These observations together suggest that in the meta-learning framework, all conformational and linear epitope predictors interact, and the degree of interaction among them differs. An ensemble of complementary base learners incorporated in a hierarchy of appropriately arranged meta learners can produce a meta classification performance that is comparable to, or superior to, the performances of the base predictors.

## Conclusions

Understanding of the interactions between antibodies and epitopes provides the basis for the rational design of preventive vaccines. Following the increased availability of protein sequences and structures, several various computational tools have been developed for epitope prediction. Our analytical and experimental results reveal the complementary performances of various epitope prediction methods, suggesting synergy among these computational tools. Unlike previous ensemble approaches, we propose a meta-learning approach for predicting B-cell epitopes, which combines base epitope prediction tools with other meta learners in a hierarchical architecture to integrate multiple predictions into meta knowledge for epitope classification. We conducted a consistent and unbiased independent test on our method, and compared the results with those from other prediction tools. Our results demonstrate that the proposed meta-learning approach outperforms the single base tools and other recently developed epitope predictors.

## Methods

### Epitope prediction as inductive learning

When addressing an inductive learning problem, by representing each example by a set of descriptive attributes, its target attribute, and the attribute values, then an inductive learning task can be defined as follows:

If

***E*** **= {*e***_***1,***_***e***_***2***_***,…,e***_***n***_**}** is a set of training examples,

***X*** **= {*x***_***1,***_***x***_***2***_***,…,x***_***m***_**}** is a set of descriptive attributes,

***C*** is the target attribute,

then each training example *e*_*i*_ is represented by a vector < *v*_*1*_*,v*_*2*_*,…,v*_*m,*_*t*_*i*_>, where *v*_*1*_*,v*_*2*_*,…,v*_*m*_ denotes a legal value of attribute *x*_*1,*_*x*_*2*_*,…,x*_*m*_, and *t*_*i*_ is a legal value of the target attribute *c*.

Assuming

***F*::*X*** **→** ***c*** is the target attribute function, which maps an example represented by a vector of descriptive attribute values to its target attribute value, and

***H*::*X*** **→** ***c*** is a hypothesis that approximates the target attribute function, *H*(*X*) ≈ *F*(*X*),

then for a test example ***t***, the target value is predicted as *H*(***t***).

Considering epitope prediction as an inductive learning problem, when provided a set of antigens with known epitopic and nonepitopic regions, the initial goal is to train a classifier from a set of antigens, each of which is described by a set of protein features, and then apply the classifier to novel antigens for epitope detection. Several learning-based epitope prediction tools have been developed [5–13]. Because different learning algorithms employ different knowledge representations and search heuristics, they explore different hypothesis space and consequently obtain different results. We propose combining multiple prediction methods to achieve superior performance compared with that achieved using a single predictor.

### Meta learning: stacked generalization and cascade generalization

Stacked and cascade generalizations are methods of combining the predictions of multiple learning models that have been trained for a classification task [14–17]. Unlike approaches based on bagging [29] or boosting [30], which aim to reduce the variance of multiple learners to improve performance, stacked and cascade generalizations both work as layered processes with the aim of reducing learner bias.

In stacked generalization, each of a set of base learners is trained in a data set, and the predictions of these base learners become the meta features. A successive layer of meta learners receives the meta features as the input with which to train the meta models in parallel, passing their output to the subsequent layer. A single classifier at the top level makes the final prediction. Stacked generalization is considered a form of meta learning because the transformations of the training data for the successive layers contain the information of the predictions of the preceding learners, which is a form of meta knowledge.

Similar to stacked generalization, cascade generalization is a form of meta learning [16,17]. Cascade generalization is distinguishable from stacked generalization because it produces a sequential, rather than a parallel, composition of classifiers in a hierarchy. Only one learner exists at each level, and its prediction becomes a novel feature, in addition to the base features, of the input to the learner in the successive level. Stacked generalization combines the predictions of multiple learners in parallel at each level in a layered architecture to improve classification accuracy, whereas cascade generalization connects multiple learners in a sequential fashion to obtain a meta model by propagating the prediction of the learner, as a novel feature, to the subsequent learner.

In this study, we developed multilevel architectures for stacked and cascade generalizations. We used C4.5 [20], k-NN [21], ANN [22], and SVM [19] as the meta learners because C4.5 learns comprehensible decision trees, the nearest-neighbor rule is capable of constructing local approximations to the target, artificial neural network learning methods provide a robust approach to approximating a wide variety of target functions, and SVM has demonstrated promising performances in various applications. We selected several state-of-the-art linear and conformational epitope prediction tools as the candidate B-cell epitope base learners, including BepiPred [5], ABCpred [6], AAP [7], BCPREDS [8], DiscoTope 2.0 [9], ElliPro [10], SEPPA 2.0 [11], and Bpredictor [18]. We analyzed and compared the base features exploited by previous prediction methods, and selected those that characterize physicochemical propensities and structural properties. We adopted 14 base features: epitope propensity [9], secondary structure [26], residue accessibility [31], B factor [32,33], solvent-excluded surfaces, solvent-accessible surfaces [34], protein chain flexibility [35], hydrophilicity [27], PSSM [36], atom volume [37], accessible surface area [38,39], side chain polarity [40], hydropathy index [41], and antigenic propensity [42]. Table 14 lists descriptions of these features. In the training stage, the outputs of the base learners and base features are passed to meta learners at higher levels to train a meta model for classification. In the prediction stage, the trained meta classifier predicts the epitopes for a previously unseen antigen protein based on the predictions of the base learners and the base features of the protein.

**Table 14:** **Summary of base features**

**Base feature**	**Description**	**Reference**
**Propensity score**	The propensity score is derived from a scoring function that sums the log-odd ratios of the amino acids in the spatial neighborhood (defined in [9]) around each residue in a given protein.	[9]
**Residue accessibility**	Using NACCESS to calculate the accessibilities of the whole molecule submitted in a pdb file. NACCESS calculates the atomic accessible surface defined by rolling a probe around a van der Waals surface. The residue accessibilities are categorized into 4 classes: all-polar, nonpolar, total-side, and main-chain.	[31]
**Secondary structure**	Secondary structure refers to highly regular local sub-structures defined by patterns of hydrogen bonds between the main-chain peptide groups.	[26]
	In such cases, the chain of amino acids folds into regular repeating structures, such as α helix, β structure, and coil.	
**Accessible surface area**	Calculated using Gerstein et al.’s calc-surface program to measure the accessible surface area of a sphere, on each point of which the center of a solvent molecule can be placed in contact with this atom without penetrating any other atoms of the molecule.	[38,39]
**Atom volume**	Calculated using Gerstein et al.’s calc-volume program. It calculates volumes by applying a geometric construction called Voronoi polyhedra to divide the total volume among the atoms in a protein model.	[37]
**B factor**	The B factor is also known as the Debye-Waller factor or the temperature factor. It is used to describe the attenuation of x-ray scattering or coherent neutron scattering caused by thermal motion. Two B factors of a protein were considered in this study: the B factor of side chain and the B factor of main chain.	[32,33]
**Solvent excluded surface**	Calculated using Sanner et al.’s MSMS program, which builds the solvent excluded surface based on the reduced surface.	[34]
**Solvent accessible surface**	Calculated using Sanner et al.’s MSMS program, which builds the solvent accessible surface based on the reduced surface.	[34]
**PSSM**	Using PSI-BLAST to search the non-redundant protein database, and derive the information content from a position specific scoring matrix as the base feature.	[36]
**Side chain polarity**	The 20 amino acids were divided into four categories: polar, nonpolar, acidic polar, and basic polar.	[40]
**Hydropathy index**	Kyte and Doolittle devised the hydopathy index by applying a sliding-window strategy that continuously determined the average hydopathy in a window as it advanced through the sequence.	[41]
**Antigenic propensity**	Kolaskar and Tongaonkar analyzed 156 antigenic determinants (<20 residues per determinant) in 34 different proteins to obtain the antigenic propensities of amino acid residues.	[42,43]
**Flexibility**	Karplus and Schulz developed the flexibility scale based on the mobility of the protein segments on 31 proteins with known structures.	[35]
**Hydrophilic scale**	Parker et al. developed the hydrophilic scale based on the high-performance liquid chromatography (HPLC) peptide retention data.	[26]

### Analysis of prediction performances: data sets and performance measures

An epitope prediction server must be trained to obtain its prediction model before it can make a prediction. Because the epitope predictors used in our study were web-based servers or software packages, they could not be retrained using novel training data. To conduct a consistent and unbiased comparative analysis of the prediction performances of these servers, we created an independent data set of antigens with known epitopes. We collected the test data sets used in DiscoTope 2.0 [9], SEPPA 2.0 [11], and Bpredictor [18], and combined them with the data of the Epitome database [44] and Immune Epitope Database (IEDB) [45] to obtain 272 antigen protein 3D structures. After removing the duplicate proteins, we obtained 246 structures. We filtered out the antigens without epitope residues annotated in Epitope Information and B cell Assay Information in the IEDB, or previously used to train the base learners to build an independent data set of 15 antigens for prediction performance evaluation (Table 15). To ensure fair comparison between different prediction methods, we used the 15 antigens with the epitope residues annotated in the IEDB on March 4, 2014 for testing, and selected 94 antigens previously used to train the base learners (Table 16) to train the classification models. We used FATCAT [46] to measure the pairwise structural dissimilarities in the training and the test antigens. The pairwise root mean squared deviation (RMSD) ranged between 0.10 and 9.75 angstroms in the training data, and between 0.10 and 9.75 in the test data. The average pairwise RMSD of the antigens in the training and the test data sets were 2.90 ± 1.12 and 3.12 ± 1.29 angstroms, respectively. The antigen protein 3D structures were used as input for the structure-based classifiers, and the corresponding antigen sequences were sent to the sequence-based predictors as input.

**Table 15:** **Independent test data set of 15 protein antigens**

1BZQ_A	1J5O_B	1KXT_A	1KXV_A	1N5Y_B	1N6Q_B	2OZ4_A	2R4R_A	2R4S_A	2VIS_C
2VIT_C	2ZJS_Y	3BSZ_F	3KJ4_A	3KJ6_A	-	-	-	-	-

**Table 16:** **Training data set of 94 protein antigens**

1A2Y_C	1ADQ_A	1AFV_A	1AHW_C	1AR1_B	1BGX_T	1BQL_Y	1BVK_C	1C08_C	1DQJ_C
1DZB_X	1DZB_Y	1EGJ_A	1EO8_A	1EZV_E	1FDL_Y	1FNS_A	1FSK_A	1G7H_C	1G7I_C
1G7J_C	1G7L_C	1G7M_C	1G9M_G	1G9N_G	1GC1_G	1HYS_B	1IC4_Y	1IC5_Y	1IC7_Y
1J1O_Y	1J1P_Y	1J1X_Y	1JHL_A	1JPS_T	1JRH_I	1KIP_C	1KIQ_C	1KIR_C	1KYO_E
1LK3_A	1MEL_L	1MHP_B	1MLC_E	1N8Z_C	1NBY_C	1NBZ_C	1NDG_C	1NDM_C	1NSN_S
1OAK_A	1ORS_C	1OSP_O	1QLE_B	1R3K_C	1RJL_C	1RVF_1	1RVF_2	1RVF_3	1RZJ_G
1RZK_G	1TZH_V	1TZI_V	1UA6_Y	1UAC_Y	1UJ3_C	1V7M_V	1W72_A	1WEJ_F	1XIW_A
1YJD_C	1YQV_Y	1YY9_A	1ZTX_E	2AEP_A	2ARJ_Q	2B2X_A	2DD8_S	2EIZ_C	2HMI_B
2Q8A_A	2QQK_A	2QQN_A	2UZI_R	2VH5_R	2VXQ_A	2VXT_I	2W9E_A	2XTJ_A	2ZUQ_A
3G6D_A	3GRW_A	3O0R_B	3PGF_A	-	-	-	-	-	-

We evaluated prediction performances by using several measures: TP rate (i.e., sensitivity), FP rate, precision (i.e., positive predictive value), percentage accuracy, F-score, and MCC. Table 17 lists the definitions of these measures. We considered a predicted antigenic residue a TP if it was within a known epitopic region. Otherwise, we considered it a FP. We considered a predicted nonantigenic residue a true negative (TN) if it was outside the known epitopes, or a false negative (FN) if it was part of a known epitope. We tested the prediction models on the independent antigen data. According to the output of the prediction models, for each amino acid we obtained: (1) the epitope prediction score, or (2) the classification (e.g., epitope or nonepitope based on a prespecified score threshold). The numbers obtained for TP, TN, FP, and FN depended on the manner in which the threshold was selected, and provided performance information. To enable the prediction models that require appropriate parameter settings to produce the optimal performances, we performed a systematic search, such as a sequential or a grid search [23], to identify the optimum parameter values for the models, and used these values to evaluate performances in subsequent experiments. Because we could retrain the meta learners, such as SVM, we conducted a grid search for the parameter values of the best-performing meta learner in CV. In contrast to the meta learners, the retraining of the base learners, such as SEPPA 2.0, was infeasible. To optimize the parameters of a base learner, alternatively we experimented the base learner on the training data set of 94 antigens (Table 16), varying the parameter values, and selected the values that produced the maximum performance. For example, to identify the scoring threshold of the best-performing SEPPA 2.0, we tested the values (0.01, 0.02, 0.03,…, 0.99) between 0 and 1 as candidate thresholds, and found 0.21 to produce the optimum performance (Table 5). Though this strategy is a naïve exhaustive search, it is effective, and can be easily parallelized [23] to improve the efficiency. In general, correlation exists between the TP rate and the FP rate produced by the predictive model. Typically, the FP rate increases with the TP rate. We prepared ROC curves to summarize the results on the different thresholds and calculated the AUC.

**Table 17:** **Definitions of performance measures**

**Performance measure**	**Definition**
TPR^a^	TP/(TP + FN)
FPR	FP/(FP + TN)
Precision^b^	TP/(TP + FP)
Accuracy	(TP + TN)/(TP + TN + FP + FN)
F-score	2 × TPR × Precision/(TPR + Precision)
MCC	$\frac{TP\times TN-FP\times FN}{\sqrt{\left(TP+FP\right)\left(TP+FN\right)\left(TN+FP\right)\left(TN+FN\right)}}$
AUC	Area under the ROC curve

^a^True Positive Rate is also known as Sensitivity or Recall.

^b^Precision is also known as Positive Predictive Value.

### Correlation analysis and ablation study

A meta classifier can consist of an arbitrary number of base learners, and its overall performance depends on these learning components. If the learning components have complementary predictive strengths, a meta classifier can search a variety of hypotheses in the hypothesis space, and provide superior generalizations for novel test data than a single-component learner can [14,17]. We used statistical techniques to analyze the prediction tools. We evaluated the correlations between the prediction scores, and between the rankings of the prediction scores. Using a Pearson’s correlation analysis, we measured the strength of the relationship between the prediction scores produced by the tools. We ranked the prediction scores produced by the tools, and calculated the Spearman’s rank correlation coefficient to investigate the correlations between the prediction score rankings of the prediction tools. The results from correlation analysis provided a basis for selecting the appropriate base learners in meta learning.

In addition to assessing the complementary prediction strengths of the prediction methods by using statistical techniques, we conducted an ablation study to measure the contribution of the base learners to the meta classifier. A meta classifier *M* is constructed from a set of base learners *A* = {*b*_*1*_, *b*_*2*_, *b*_*3*_,…,*b*_*m*_}. We denoted the meta classifier as *M*(*A*), and its performance depended on the behaviors of the base learners selected. Given a set of available base learners *B* = {*b*_*1*_, *b*_*2*_, *b*_*3*_,…,*b*_*n*_}, where *n* ≥ *m*, an exponential number of possible selections could derive from these base learners to develop a particular meta learner. To reduce the computational costs of the ablation analysis, we adopted two greedy iterative approaches, backward elimination and forward selection, to evaluate the contributions of available base learners. The greedy iterative backward elimination approach started with the maximal meta classifier built upon all available base learners in *B* (i.e. *M*(*A*) = *M*(*B*)). In each iteration, it performed the following tasks:

1. Identified *b*_*i*_ ∈ *B*, so that the prediction performance of *M*(*B*\{*b*_*i*_}) is the worst, where *B*\{*b*_*i*_} indicates *B* without *b*_*i*_.
2. Removed *b*_*i*_ from *B*.
3. If |*B*| ≥1, returned to step (1) and iterated, or stopped.

By contrast, the greedy iterative forward selection approach started with the minimal classifier built without any base learner in *B* (i.e. *M*(*A*) = {}). In each iteration, it performed the following tasks:

1. Identified *b*_*i*_ ∈ *B*, so that the prediction performance of *M*(*A*∪{*b*_*i*_}) is the best, where *A*∪{*b*_*i*_} indicates *A* with *b*_*i*_ added.
2. Removed *b*_*i*_ from *B*.
3. If |*B*| ≥1, returned to step (1) and iterated, or stopped.

We compared the relevance of the base learners to the meta classifier by the order of their removal or addition, and estimated their effects on the meta classification by the amount of decrease or increase in prediction performance.

### Availability

The data (training and test) and the executable code of the meta classifiers (stacking and cascade) are available. In the Additional file 1, we showed the link to the data and the code, and described the instructions for executing the code and the procedures for preparing the data. The user can run the meta classifiers that have been trained on the training data to predict the B-cell epitopes on protein antigens.

## Additional file

Additional file 1:
**Supplement_BMC.**

## References

[1] Hopp TP, Woods KR (1981). Prediction of protein antigenic determinant from amino acid sequences. Proc Natl Acad Sci U S A.

[2] Pellequer J, Westhof E, Van Regenmortel M (1993). Correlation between the location of antigenic sites and the prediction of turns in proteins. Immunol Lett.

[3] Pellequer J, Westhof E (1991). Predicting location of continuous epitopes in proteins from their primary structures. Meth Enzymol.

[4] Blythe MJ, Doytchinova IA, Flower DR (2002). JenPep: a database of quantitative functional peptide data for immunology. Bioinformatics.

[5] Larsen JE, Lund O, Nielsen M (2006). Improved method for predicting linear B-cell epitopes. Immunome Res.

[6] Saha S, Raghava G (2006). Prediction of continuous B-cell epitopes in an antigen using recurrent neural network. Proteins.

[7] Chen J, Liu H, Yang J, Chou K (2007). Prediction of linear B-cell epitopes using amino acid pair antigenicity scale. Amino Acids.

[8] El-Manzalawy Y, Dobbs D, Honavar V (2008). Predicting linear B-cell epitopes using string kernels. J Mol Recognit.

[9] Kringelum JV, Lundegaard C, Lund O, Nielsen M (2012). Reliable B cell epitope predictions: impacts of method development and improved benchmarking. PLoS Comput Biol.

[10] Ponomarenko J, Bui HH, Li W, Fusseder N, Bourne PE, Sette A, Peters B (2008). ElliPro: a new structure-based tool for the prediction of antibody epitopes. BMC Bioinformatics.

[11] Qi T, Qiu T, Zhang Q, Tang K, Fan Y, Qiu J, Wu D, Zhang W, Chen Y, Gao J, Zhu R, Cao Z (2014). SEPPA 2.0-more refined server to predict spatial epitope considering species of immune host and subcellular localization of protein antigen. Nucleic Acids Res.

[12] Rubinstein ND, Mayrose I, Martz E, Pupko T (2009). Epitopia: a web-server for predicting B-cell epitopes. BMC Bioinformatics.

[13] Liang S, Zheng D, Standley DM, Yao B, Zacharias M, Zhang C (2010). EPSVR and EPMeta: prediction of antigenic epitopes using support vector regression and multiple server results. BMC Bioinformatics.

[14] Wolpert DH (1992). Stacked generalization. Neural Netw.

[15] Ting KM, Witten IH (1997). Stacked Generalization: When Does it Work?.

[16] Gama J (1998). Combining Classifiers by Constructive Induction.

[17] Gama J, Brazdil P (2000). Cascade generalization. Mach Learn.

[18] Zhang W, Xiong Y, Zhao M, Zou H, Ye X, Liu J (2011). Prediction of conformational B-cell epitopes from 3D structures by random forests with a distance-based feature. BMC Bioinformatics.

[19] Chang CC, Lin CJ (2011). LIBSVM: A library for support vector machines. ACM Trans Intelligent Systems and Technology.

[20] Quinlan JR (1993). C4.5: Programs for Machine Learning.

[21] Duda RO, Hart PE, Stork DG (2001). Pattern Classification.

[22] Bishop CM (1996). Neural Networks for Pattern Recognition.

[23] Hsu CW, Chang CC, Lin CJ: **A practical guide to support vector classification.** 2010, Tech Report, Dept Computer Science and Information Engineering, National Taiwan University http://www.csie.ntu.edu.tw/~cjlin/papers/guide/guide.pdf.

[24] Ansari HR, Raghava GPS (2010). Identification of conformational B-cell Epitopes in an antigen from its primary sequence. Immunome Res.

[25] Singh H, Ansari HR, Raghava GPS (2013). Improved method for linear B-cell epitope prediction using Antigen’s primary sequence. PLoS ONE.

[26] Nagano K (1973). Logical analysis of the mechanism of protein folding: I. predictions of helices, loops and beta-structures from primary structure. J Mol Biol.

[27] Parker JM, Guo D, Hodges RS (1986). New hydrophilicity scale derived from high-performance liquid chromatography peptide retention data: correlation of predicted surface residues with antigenicity and X-ray-derived accessible sites. Biochemistry.

[28] Baldi P, Brunak S, Chauvin Y, Anderson CAF, Nielsen H (2000). Assessing the accuracy of prediction algorithms for classification: an overview. Bioinformatics.

[29] Breiman L (1996). Bagging predictors. Mach Learn.

[30] Schapire R (1990). The strength of weak learnability. Mach Learn.

[31] Hubbard SJ, Thornton JM: *NACCESS Computer Program.* Department of Biochemistry and Molecular Biology, University College London; 1993.

[32] Lipkin HJ (2004). Physics of Debye-Waller Factors.

[33] Liu R, Hu J (2011). Prediction of discontinuous B-cell epitopes using logistic regression and structural information. J Proteomics Bioinform.

[34] Sanner MF, Olson AJ, Spehner JC (1996). Reduced surface: an efficient way to compute molecular surfaces. Biopolymers.

[35] Karplus PA, Schulz GE (1985). Prediction of chain flexibility in proteins: a tool for the selection of peptide antigen. Naturwissenschaften.

[36] Zhang Z, Schäffer AA, Miller W, Madden TL, Lipman DJ, Koonin EV, Altschul SF (1998). Protein sequence similarity searches using patterns as seeds. Nucleic Acids Res.

[37] Gerstein M, Tsai J, Levitt M (1995). The volume of atoms on the protein surface: calculated from simulation, using voronoi polyhedra. J Mol Biol.

[38] Lee B, Richards FM (1971). The interpretation of protein structures: estimation of static accessibility. J Mol Biol.

[39] Gerstein M (1992). A resolution-sensitive procedure for comparing protein surfaces and its application to the comparison of antigen-combining sites. Acta Cryst.

[40] Hausman RE, Cooper GM (2003). The Cell: A Molecular Approach.

[41] Kyte J, Doolittle RF (1982). A simple method for displaying the hydropathic character of a protein. J Mol Biol.

[42] Kolaskar AS, Tongaonkar PC (1990). A semi-empirical method for prediction of antigenic determinants on protein antigens. FEBS Lett.

[43] Saha S, Raghava GPS: *BcePred:Prediction of Continuous B-Cell Epitopes in Antigenic Sequences Using Physico-chemical Properties*, ICARIS 2004, LNCS 3239. New York/Munich/Milan: Springer; 197–204.

[44] Schlessinger A, Ofran Y, Yachdav G, Rost B (2006). Epitome: database of structure-inferred antigenic epitopes. Nucleic Acids Res.

[45] Ponomarenko J, Papangelopoulos N, Zajonc DM, Peters B, Sette A, Bourne PE (2011). IEDB-3D: structural data within the immune epitope database. Nucleic Acids Res.

[46] Ye Y, Godzik A (2003). Flexible structure alignment by chaining aligned fragment pairs allowing twists. Bioinformatics.

