# Supplementary material for: A meta-learning approach for B-cell conformational epitope prediction
Source: BMC Bioinformatics. 2014 Nov 18;15(1):378. doi: 10.1186/s12859-014-0378-y (PMC4237749; doi:10.1186/s12859-014-0378-y)

**Introduction**

The objective of this study of B-cell epitope prediction is twofold: (1) to analyze the complementary predictive strengths in different prediction tools, and (2) to introduce a generic computational model to exploit the synergy among various prediction tools. The computational model is based on meta learning. It has the flexibility to integrate various component prediction tools into a unique prediction system. Therefore, our goal is not to develop any particular classifier for B-cell epitope prediction, but instead we intend to advocate the applicability of meta learning to epitope prediction, and to show its performance that is comparable with (or superior to) those of other approaches.

For the purpose of demonstration, we provide the executable code of the meta classifiers (stacking and cascade) previously trained on 94 proteins for this study (see Table 13 in paper). The user can test the already trained meta classifiers to predict the B-cell epitopes on a protein antigen.

**Availability**

The data and the code used in this study can be found at <https://drive.google.com/folderview?id=0B0VnPrX_OoXgX2s4SnRwazVGZGc&usp=sharing>

**Materials**

**Computer code of the trained 2-level stacking meta classifier:**

1. svm_stack.exe: executable code of the trained level-2 meta learner (SVM)
2. svm-predict.exe: executable code of libSVM (used by svm_stack.exe)
3. lev1_stack.jar: Java code of the level-1 meta learners (C4.5, k-NN, and ANN) trained in parallel
4. lev1_stack.exe: executable code of the trained level-1 meta classifier that calls lev1_stack.jar

**Computer code of the trained 4-level cascade meta classifier:**

1. svm_cascade.exe: executable code of the trained level-4 meta learner (SVM)
2. svm-predict.exe: executable code of libSVM (used by svm_cascade.exe)
3. lev13_cascade.jar: Java code of the trained cascaded meta learners from levels 1 to 3 (k-NN, C4.5, and ANN)

**Computer code for data normalization:**

1. norm.exe: executable code to normalize the raw data (base features values and prediction tool outputs)

**Data folders/subfolders:**

1. stacking_model: a folder that stores the computational stacked learning models of the meta learners trained on 94 protein antigens (see Table 13 in paper): C4.5, k-NN, ANN, and SVM
2. cascade_model: a folder that stores the computational cascade learning models of the meta learners trained on 94 protein antigens (see Table 13 in paper): C4.5, k-NN, ANN, and SVM
3. test_data: a folder that contains 23 subfolders of the descriptions about the 15 test proteins, including the base feature values, and the outputs of the 8 prediction tools. To predict epitopes, the meta classifiers only use the information of the base features, and the outputs of the 8 tools. In addition, the epitope locations annotated in the IEDB are provided in a subfolder named Real_epitopes for the user’s reference to evaluate the prediction performance.
   - 1. Subfolder “Real_epitopes” contains 15 text file, each corresponding to a test protein antigen. Each file shows the epitope identity of each amino acid (AA) according to the IEDB, where 0 denotes a nonepitope, and 1 denotes an epitope.


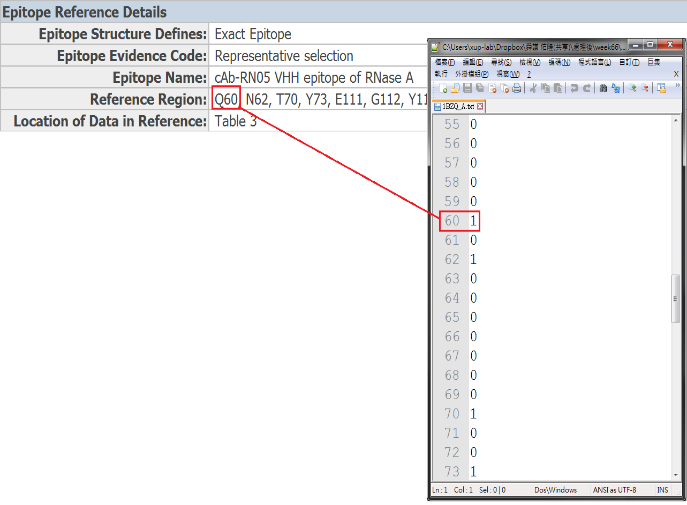


- - 1. Each of the 8 subfolders, DiscoTope, Bpredictor, SEPPA, ElliPro, ABCpred, BCPREDS, AAP, and BepiPred, contains 15 text files. Each file contains the output score for each AA on a protein antigen.
    2. The remaining subfolders contain the base feature values. Each text file contains the values of a particular base feature of the AAs on a protein antigen, e.g. hydrophilic scale. These text files are stored in the corresponding subfolders, e.g. Hydrophilic_scale or Flexibility.

1. train_data: a folder that contains 24 subfolders of the descriptions about the 94 proteins for training, including the base feature values, the outputs of the 8 prediction tools, and the epitope locations annotated in the IEDB. The information in these subfolders were used previously to train the meta classifiers, and provided here for the user’s reference.
2. normal_data: a folder that contains the normalized (z-score) training and test data used by the meta classifiers (Note. We normalized the training and the test data in advance for the user.)
3. ts.data: an input file containing a list of PDB IDs for the mata classifiers to predict the epitopes, where each single line stores only one PDB ID with a chain ID, e.g. 1BZQ_A.
4. protein_sequence: a folder that contains the sequences of the 94 training proteins and the 15 test proteins in this study


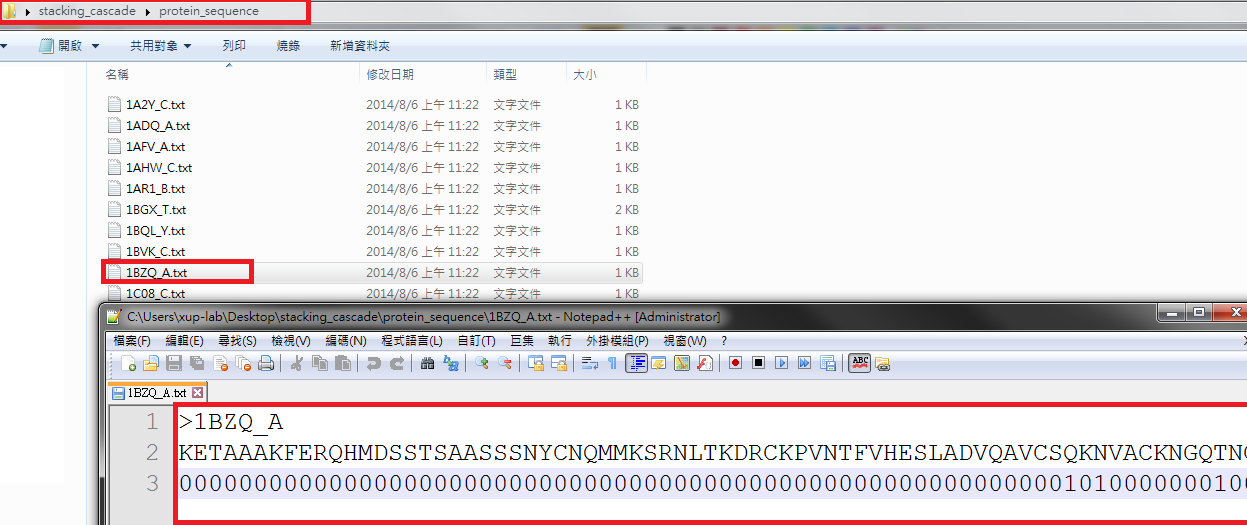


1. EpiT-0.9.rar: a compressed file that contains the AAP and the BCPREDS prediction programs

**Note 1.** The folder/subfolder names, and the file names cannot be changed because they are referred to by the meta classifiers.

**Instructions to use the trained meta classifiers to predict the epitopes on the 15 test proteins**

1. Install Java Runtime Environment for Windows systems.
2. Create a main folder, e.g. MetaClassify, where to place the code (svm_stack.exe, lev1_stack.jar, lev1_stack.exe, svm_cascade.exe, lev13_cascade.jar, and svm-predict.exe), the scripts (stacking.bat, cascade.bat) and the folders (stacking_model, cascade_model, train_data, test_data, and normal_data). This main folder will serve as a working folder where the prediction results will be placed.
3. For testing, create a ts.data file that contains a list of PDB IDs of interest from the 15 test proteins. A sample ts.data may look like the following.

In ts.data file (a text file):

1BZQ_A

1N6Q_B

3KJ4_A

1. In Windows systems:
2. How to execute the stacking meta classifier

- Double click stacking.bat to perform the stacking meta classification.

1. It first runs lev1_stack.exe and lev1_stack.jar automatically on ts.data to obtain the level-1 output results, which are stored in the subfolder stacking_lev1_output.
2. It then runs svm_stack.exe automatically on the level-1 output to obtain the level-2 output results, which are the final meta classifications, and stored in the subfolder stacking_lev2_output.


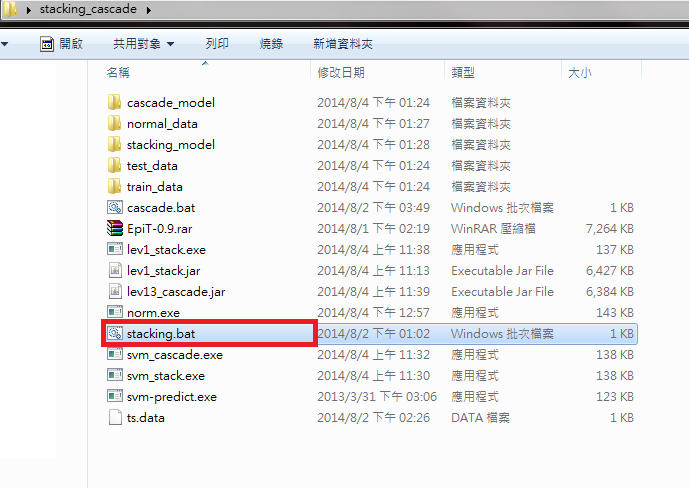


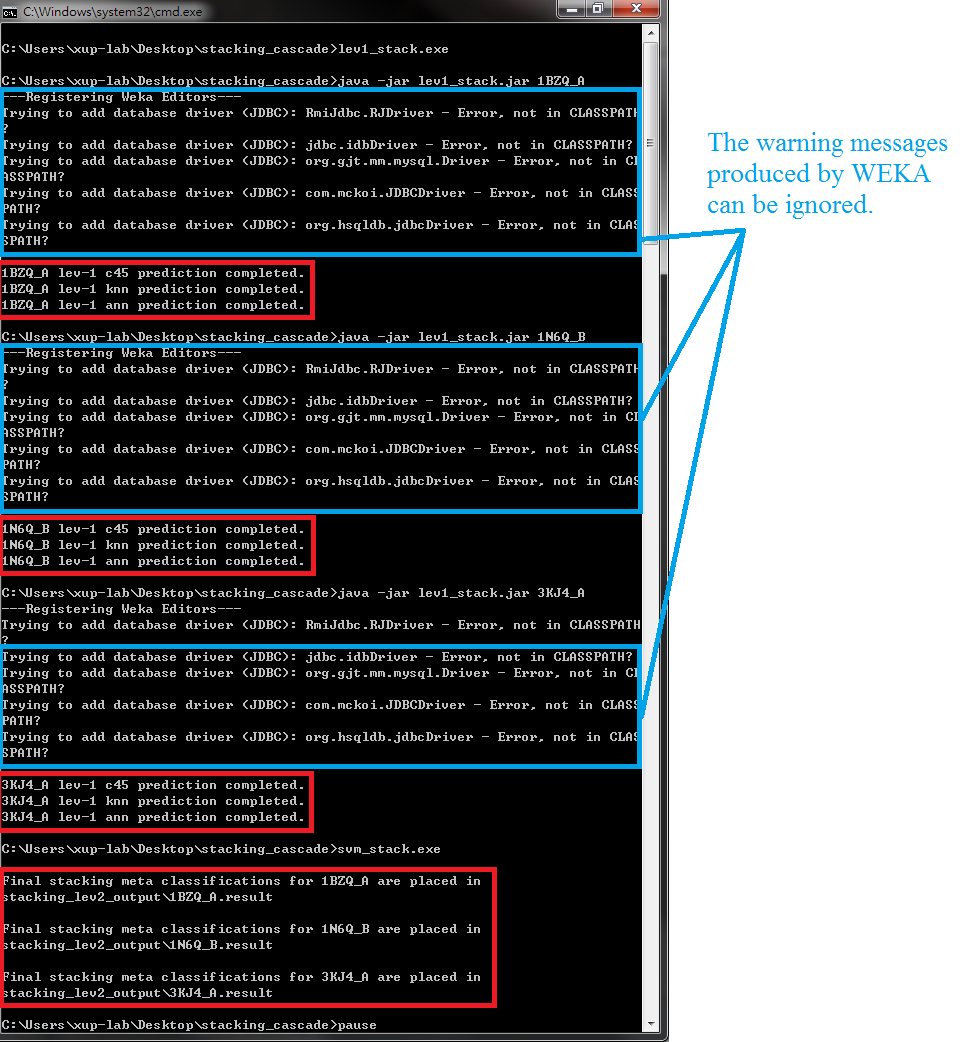


Two stacking output folders: (1) stacking_lev1_output, and (2) stacking_lev2_output


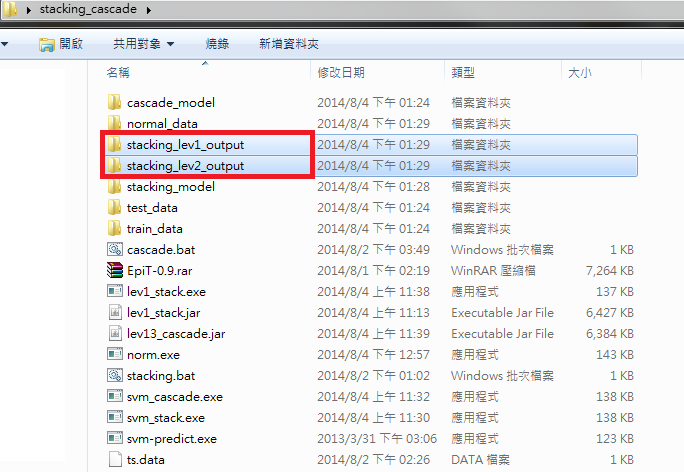


Content of stacking_lev1_output


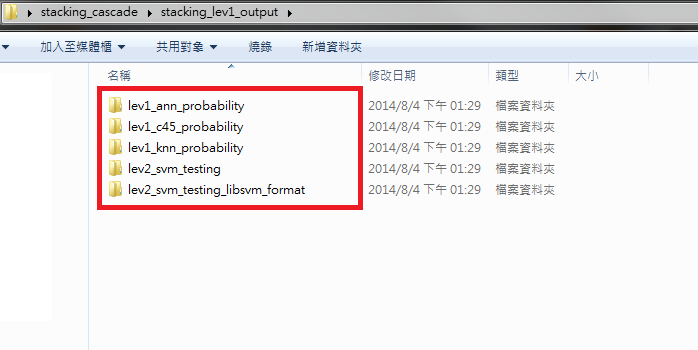


(1) Contents of stacking_lev2_output

(2) Final stacking meta classifications for 3KJ4_A


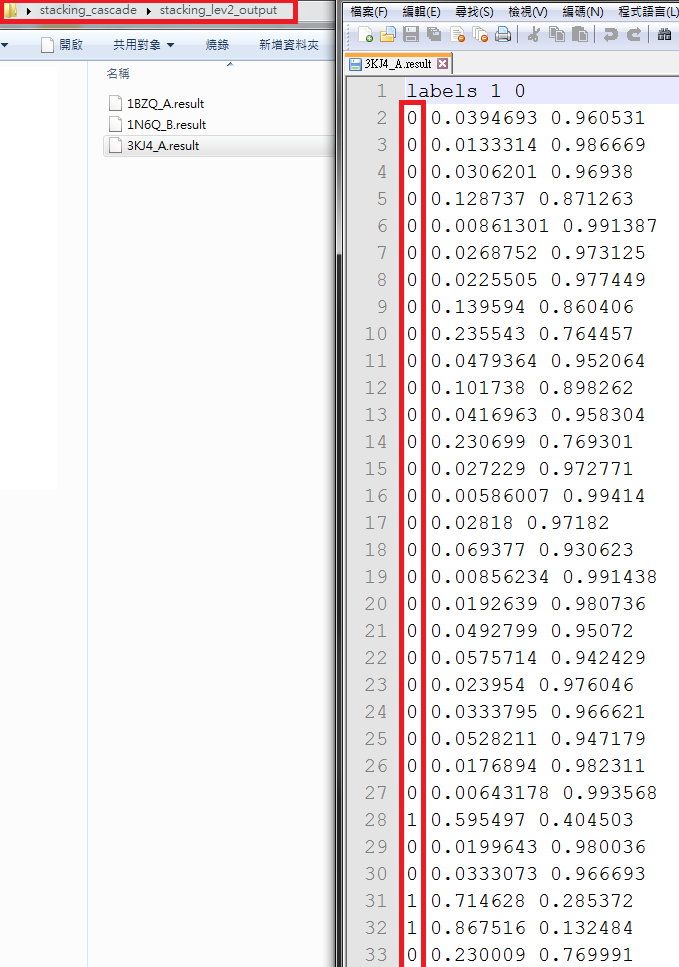


1. How to execute the cascade meta classifier

- Double click cascade.bat to perform the cascade meta classification.

1. It first run lev13_cascade.jar on ts.data to obtain the level-3 output results, which are stored in the subfolder cascade_lev13_output.
2. It then run svm_cascade.exe on the level-3 output to obtain the level-4 output results, which are the final meta classifications, and stored in the subfolder cascade_lev4_output.


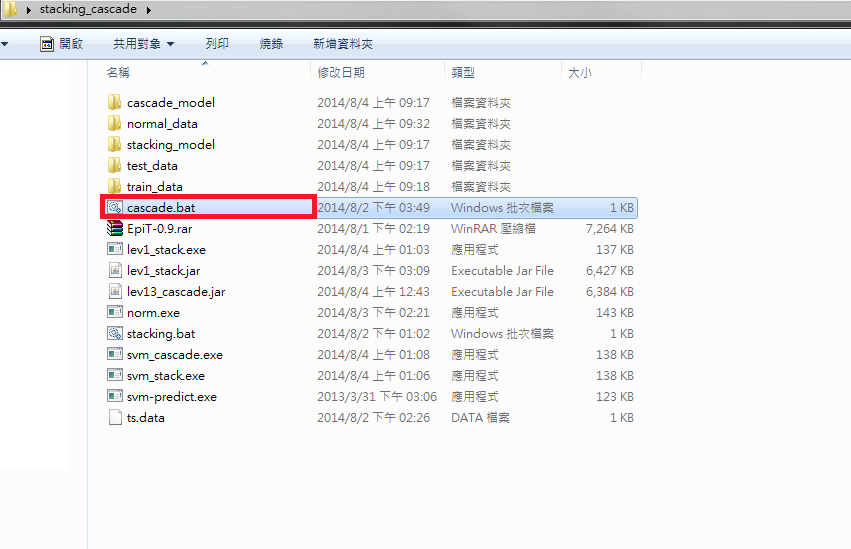

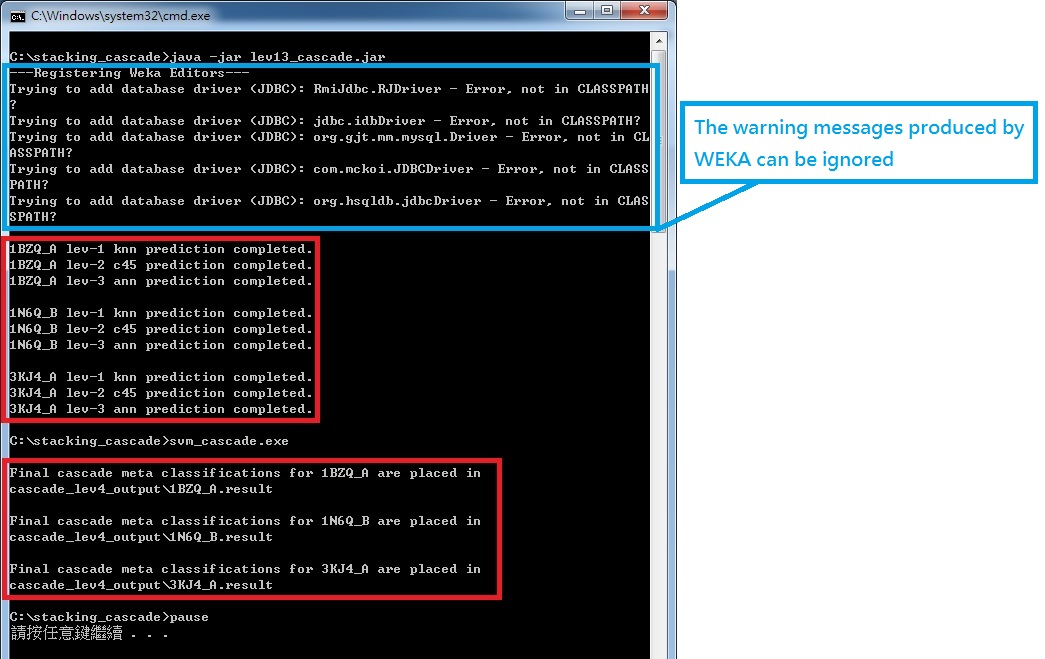


Two cascade output folders: (1) cascade_lev13_output, and (2) cascade_lev4_output


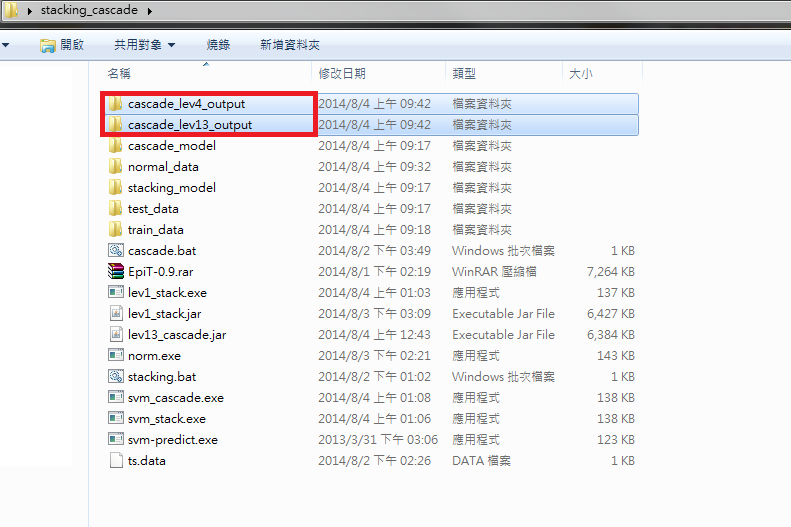


Contents of cascade_lev13_output


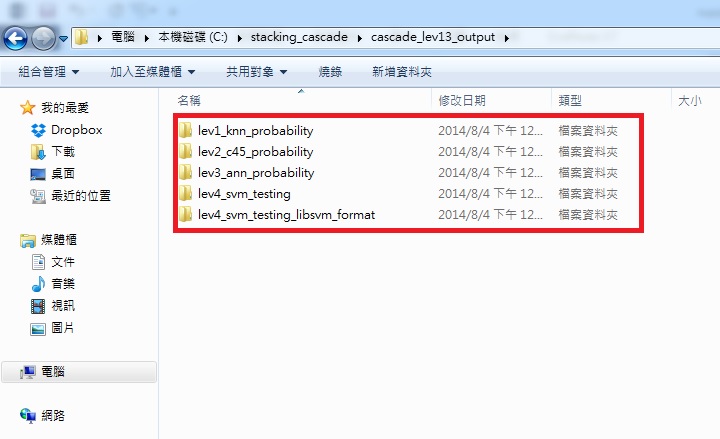


(1) Contents of cascade_lev4_output

(2) Final cascade meta classifications for 3KJ4_A


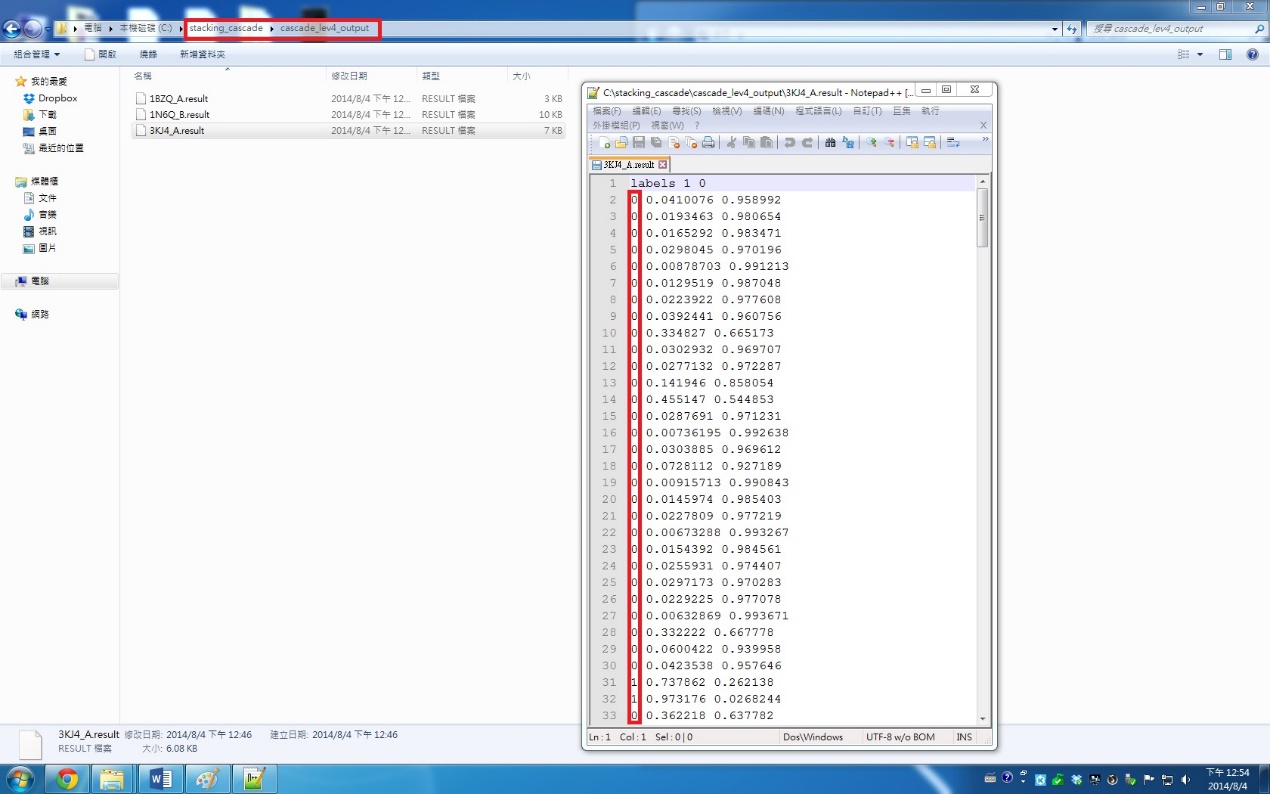


**Instructions to use the trained meta classifiers to predict the epitopes on other protein antigens**

1. Install Java Runtime Environment for Windows systems.
2. Create a main folder, e.g. MetaClassify, where to place the code (norm.exe, svm_stack.exe, lev1_stack.jar, lev1_stack.exe, svm_cascade.exe, lev13_cascade.jar, and svm-predict.exe), the scripts (stacking.bat, cascade.bat) and the folders (stacking_model, cascade_model, train_data, test_data, and normal_data). This main folder will serve as a working folder where the prediction results will be placed.
3. For testing, create a ts.data file that contains a list of PDB IDs of interest. A sample ts.data may look like the following.

In ts.data file (a text file):

1BZQ_A

1N6Q_B

3KJ4_A

1. Prepare the base feature values and the outputs of the 8 tools for the test proteins. Organize the information into text files, e.g. 1FBI_X.txt, as shown in the subfolder test_data. Each text file describes a particular feature, or a tool output for a specific protein. Place these text files in the subfolder test_data. For the same test protein, e.g. 1FBI_X, all the text files must have the same file name, but they are placed in the corresponding subfolders. For example, the text file that stores the information of hydrophilic scale must be placed in the subfolder Hydrophilic_scale. In addition, for the same protein, e.g. 1FBI_X , all the text files regarding this particular protein must have the same number of values in the files because each value corresponds to, and describes an AA of this protein.


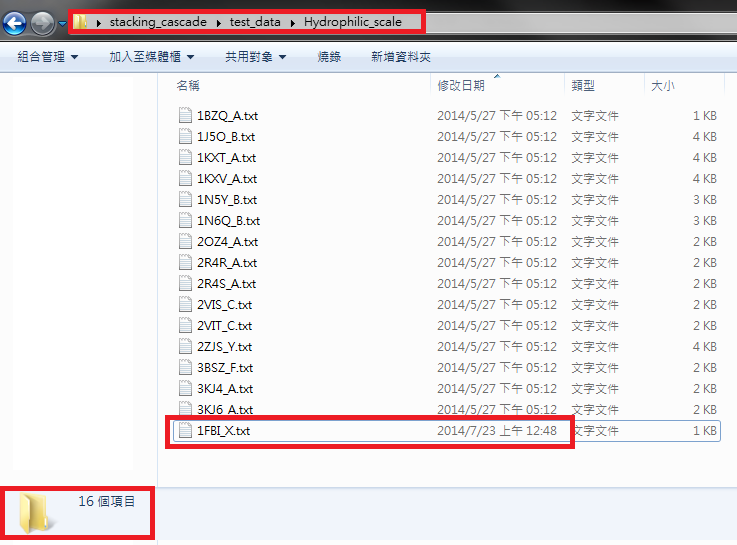


1. Normalize the test data by executing norm.exe on ts.data.

Double click norm.exe to normalize the data. The normalized data are placed in the subfolder normal_data.


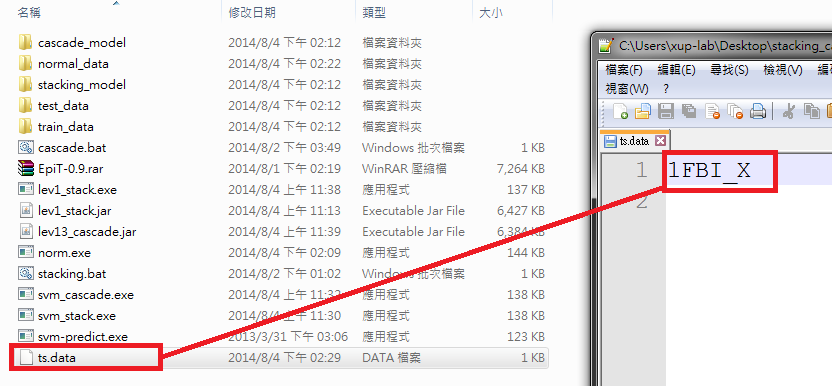


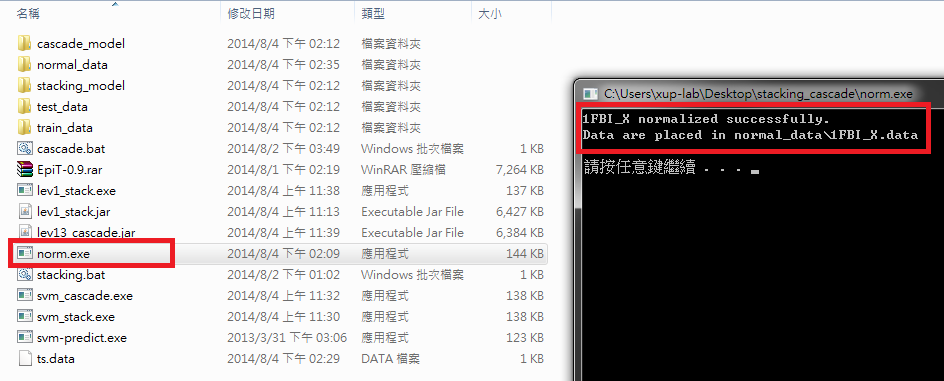


Contents of normal_data before normalization


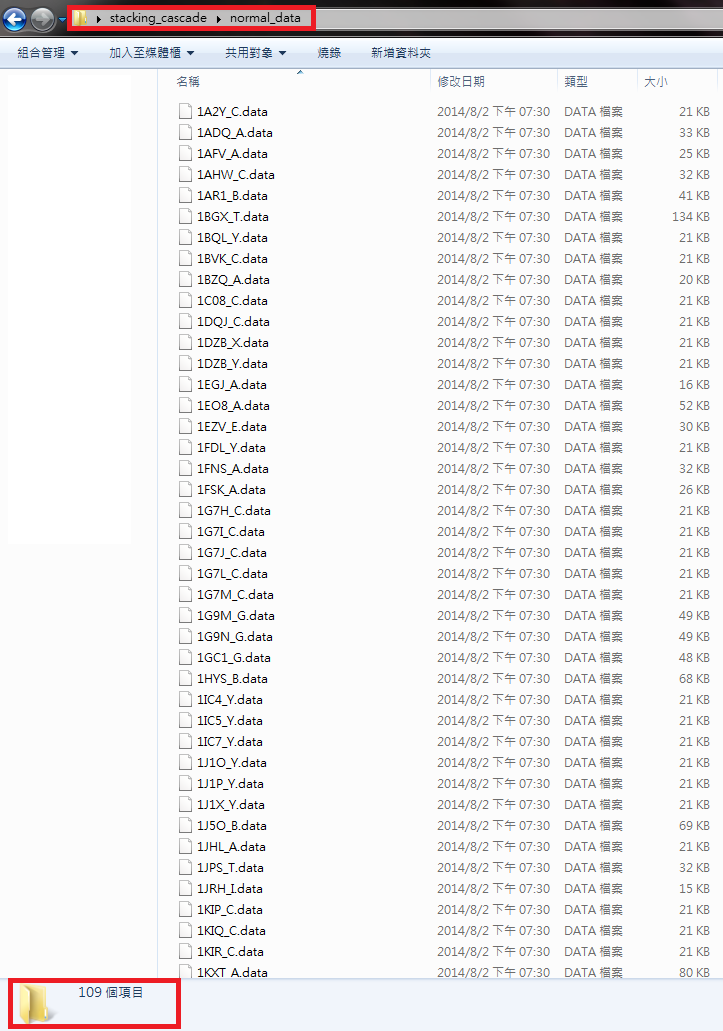


(1) Contents of normal_data after normalization

(2) Contents of 1FBI_X.data (normalized data of 1FBI_X)


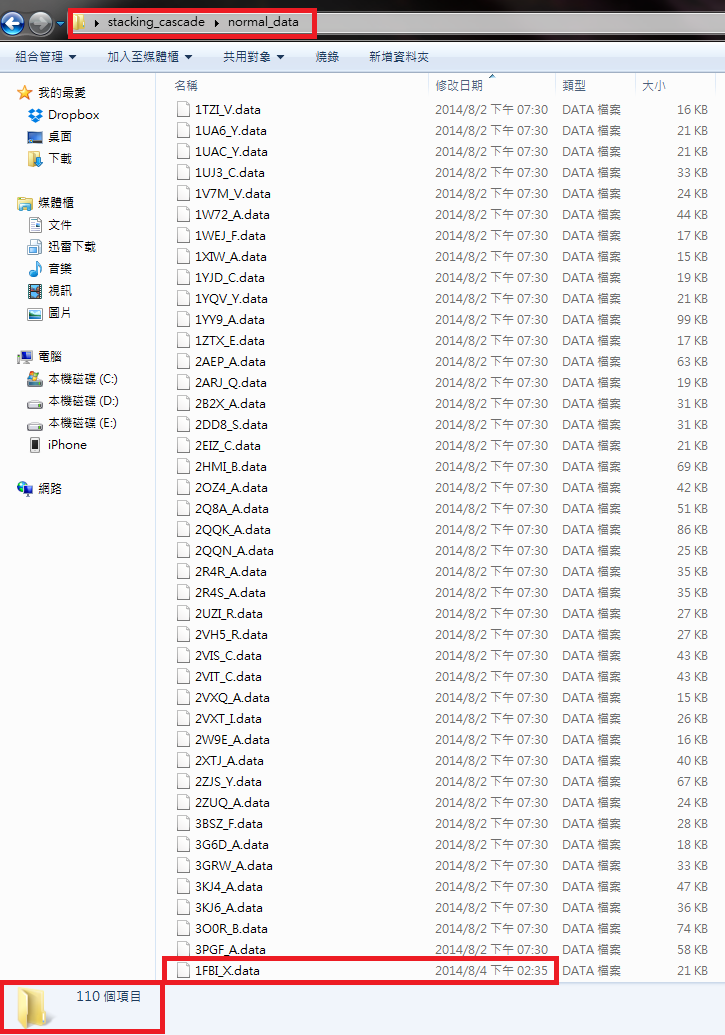


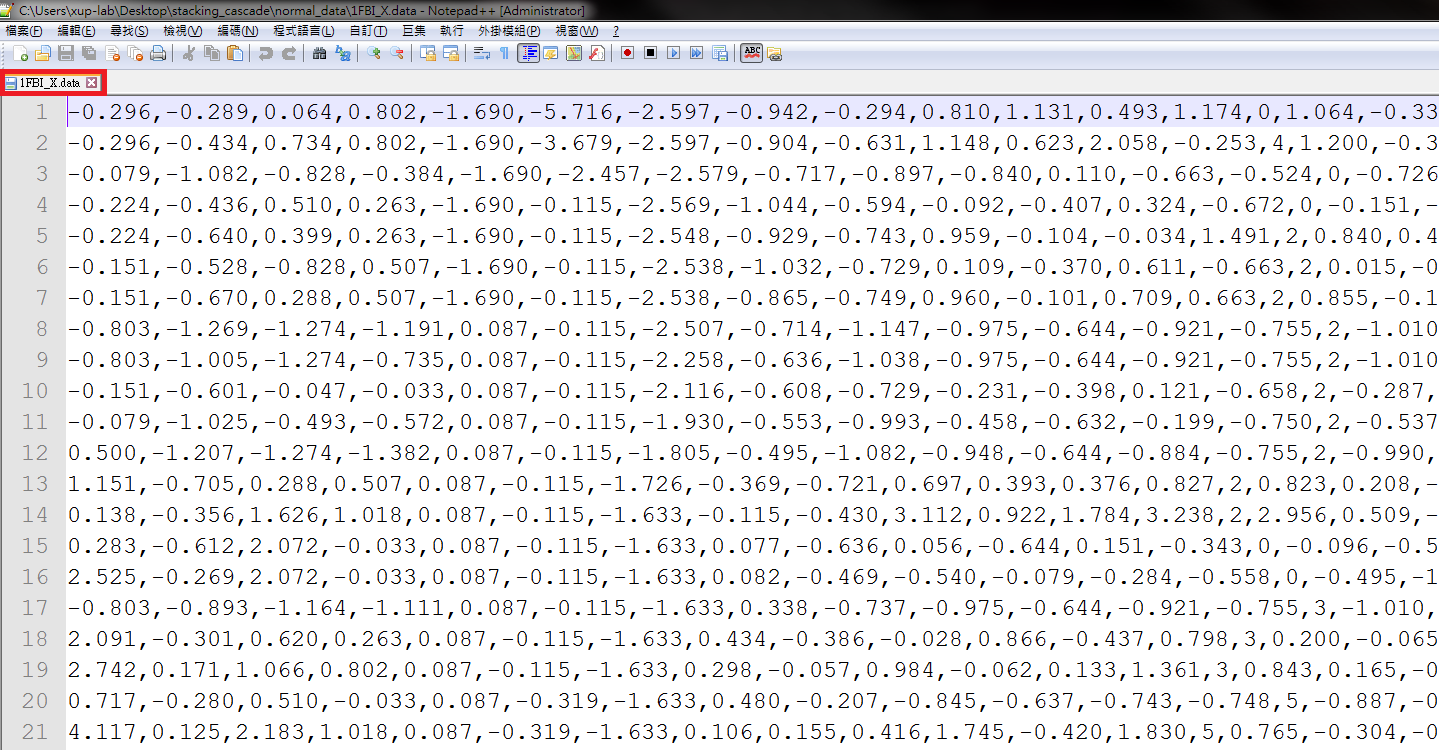


1. The following steps of executing the meta classifiers are the same as above for classifying the 15 test proteins.

**Note 2.** The meta classifiers (stacking and cascade) provided here were trained on only 94 protein antigens previously. While the prediction performance is limited to the meta knowledge learned from these 94 proteins, the experimental results demonstrated the comparable performance (see Results on the paper). When new training proteins are available, the meta classifiers can be retrained to learn more accurate meta knowledge from the new training data. The retraining of the meta classifiers involves parameter tuning required of some meta learners, e.g. SVM. Though the parameter tuning is automatically controlled by systematic search strategies (e.g. grid search), the tuning time may be significant. The computer code for the training of the meta classifiers is not provided, but available upon request.

**How to prepare test (and training) data**

1. Obtain the output from prediction tools:

From the prediction results of the tools, obtain the prediction score of each AA on the tested protein. For example, on 1BZQ, we obtain the score 0.972 for the first AA from DiscoTope’s output. Store this score in a text file 1BZQ_A.txt that is placed in the subfolder DiscoTope (within the test_data folder).

Taking 1BZQ for example, we show the snapshots of the output results, and the corresponding text file for each of the 8 prediction tool as follows.

- 1. DiscoTope2.0

<http://www.cbs.dtu.dk/services/DiscoTope/>


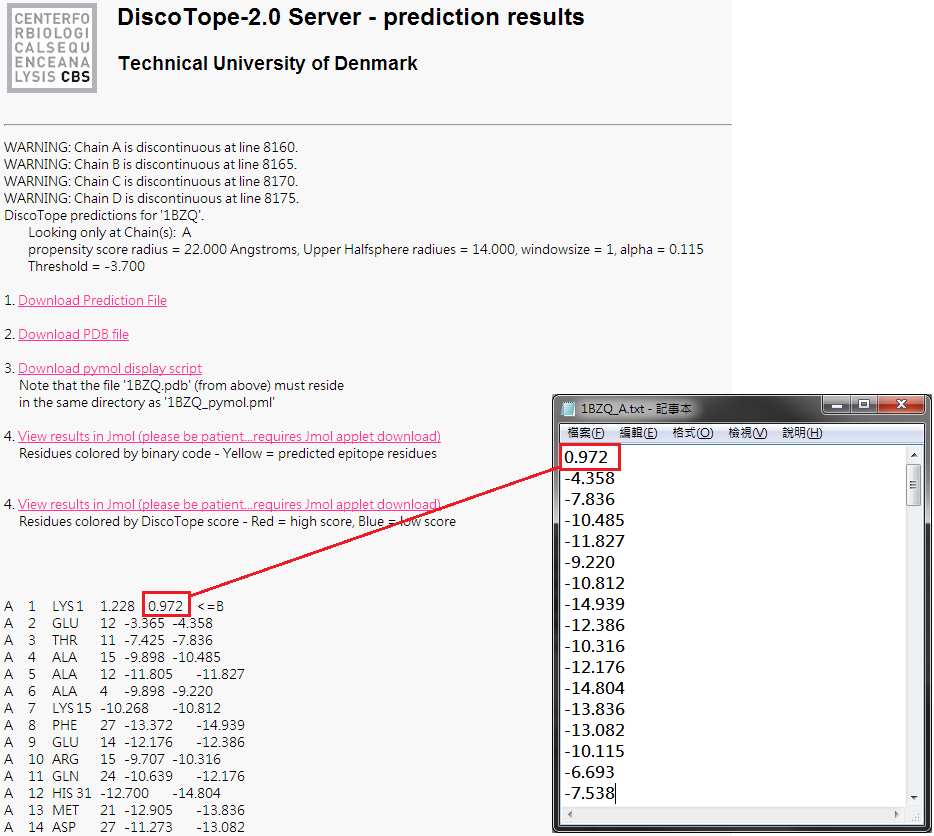


- 1. SEPPA2.0

<http://badd.tongji.edu.cn/seppa/index.php>


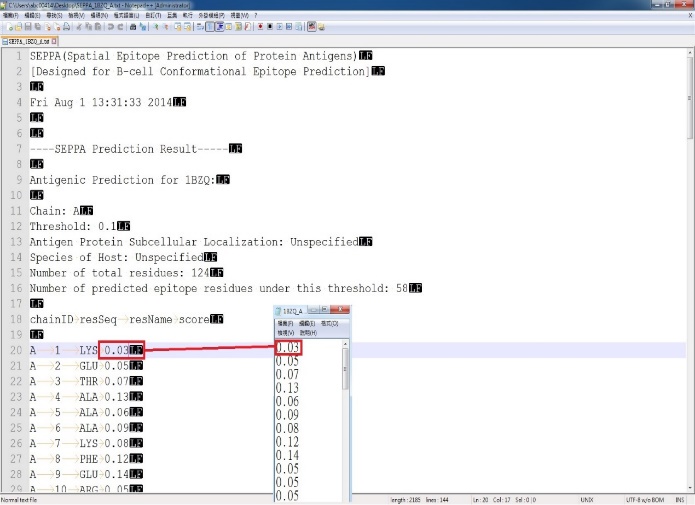


- 1. Bpredictor

<https://code.google.com/p/my-project-bpredictor/downloads/list>


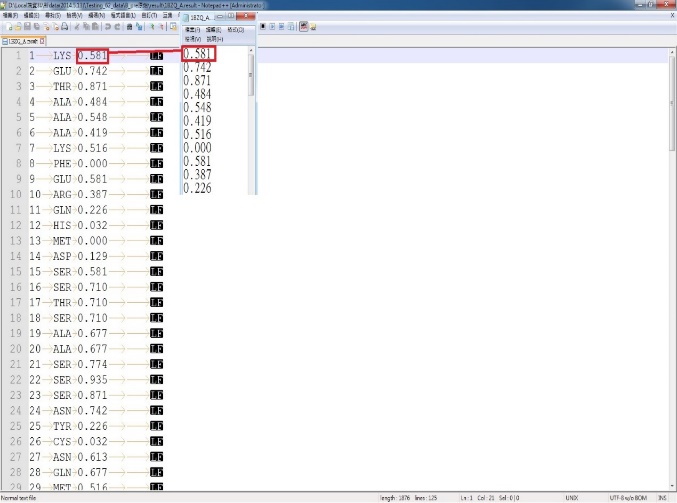


- 1. ElliPro

<http://tools.immuneepitope.org/tools/ElliPro/iedb_input>


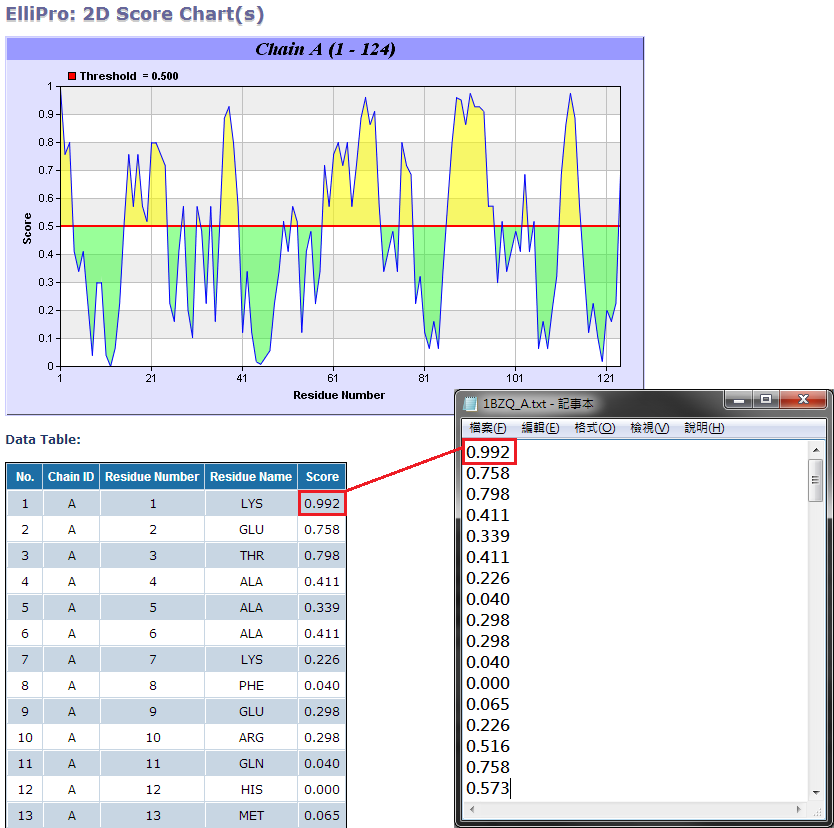


- 1. AAP

The AAP program is provided within the Epitope Toolkit. Please download EpiT-0.9.rar.

Input the protein sequence to AAP model for epitope prediction. AAP model produces the prediction scores of the overlapping protein peptides, each of a specified window size, as it slides the window along the entire protein sequence. The maximal score of an AA in the overlapping peptides is selected, and stored in a text file (e.g. 1BZQ_A.txt) for later use of the meta classifiers.


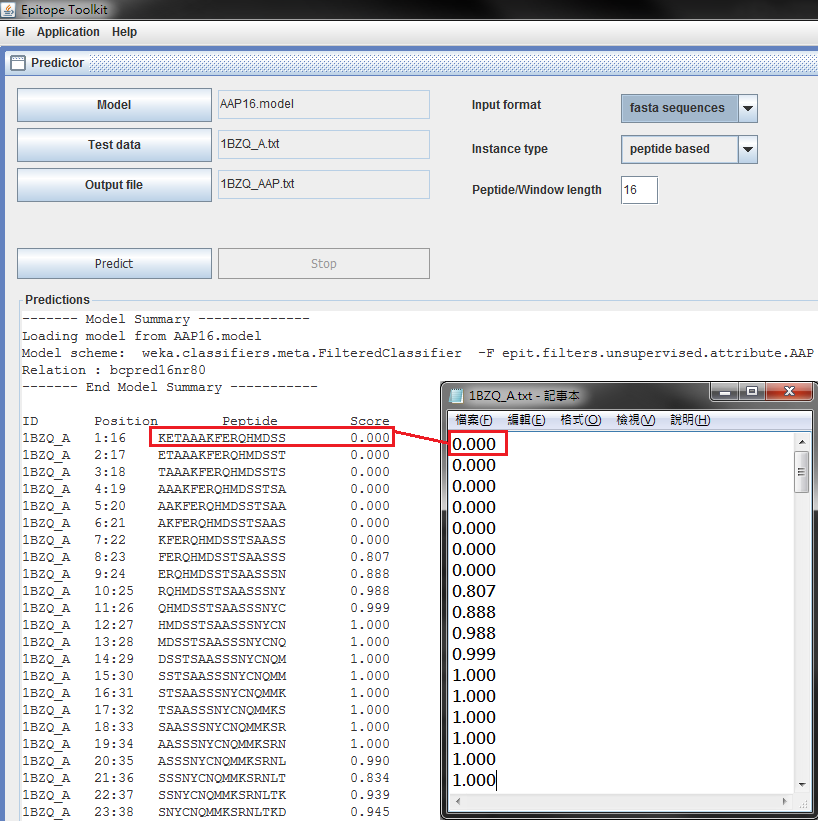


- 1. ABCpred

<http://www.imtech.res.in/raghava/abcpred/ABC_submission.html>

Input the protein sequence to ABCpred for epitope prediction. ABCpred produces the prediction scores of the protein peptides of a specified length. Assign the score to each AA on the peptide, and store the score in a text file (e.g. 1BZQ_A.txt) for later use of the meta classifiers.


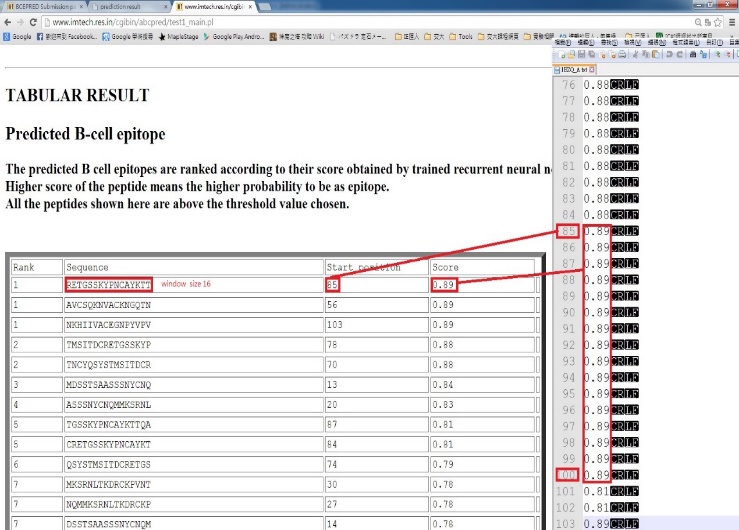


- 1. BCPREDS

The BCPREDS program is provided within the Epitope Toolkit. Please download EpiT-0.9.rar.

Input the protein sequence to BCPREDS model for epitope prediction. BCPREDS model produces the prediction scores of the overlapping protein peptides, each of a specified window size, as it slides the window along the entire protein sequence. The maximal score of an AA in the overlapping peptides is selected, and stored in a text file (e.g. 1BZQ_A.txt) for later use of the meta classifiers.


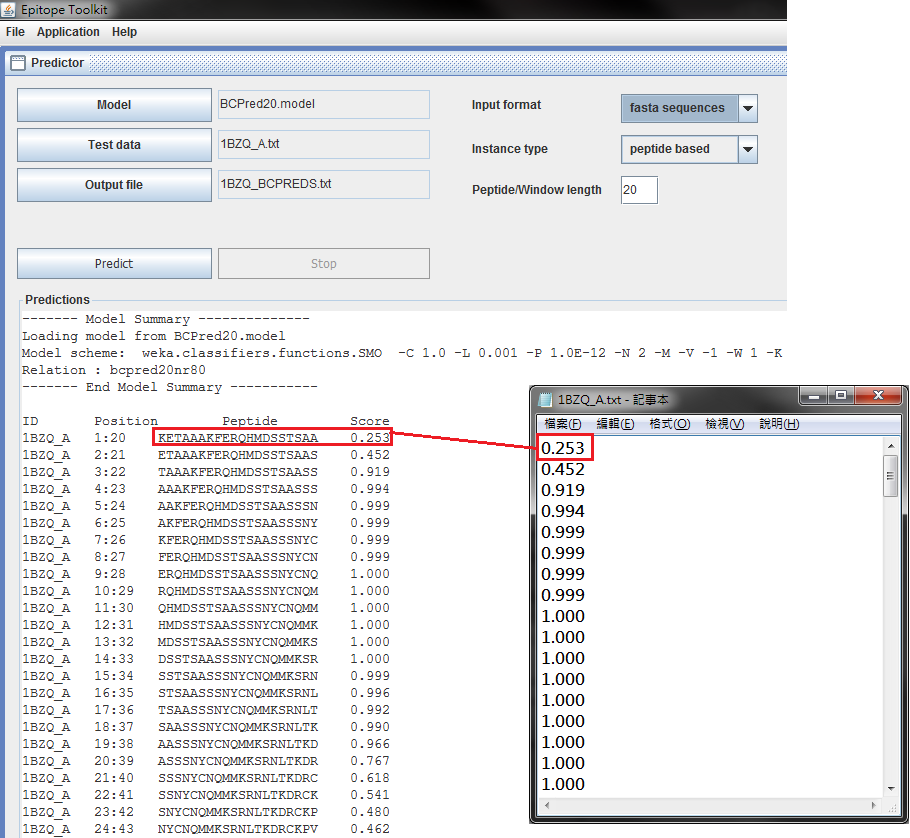


- 1. BepiPred

<http://www.cbs.dtu.dk/services/BepiPred/>


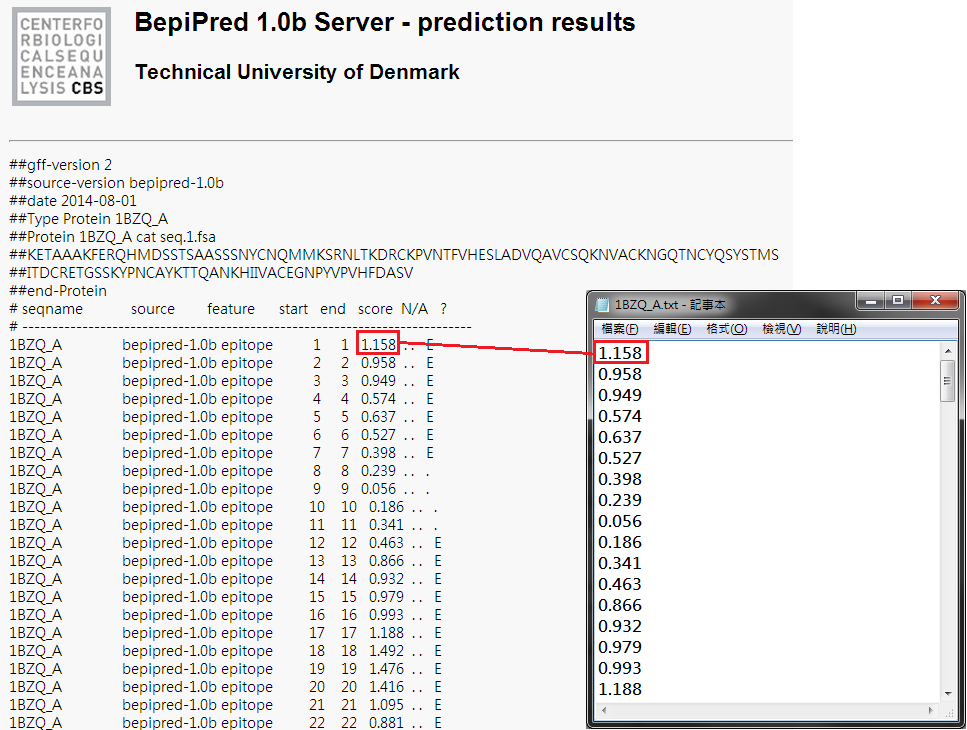


1. Obtain the values base features:

To obtain the values of the base features, use the appropriate web-based tools, e.g. NACESS. Store the base feature values in a text file, and place the file in the corresponding subfolder, e.g. Hydrophilic_scale.

- 1. Propensity score

In addition to an epitope prediction score of each AA, DiscoTope also produces a propensity score, which is used as a base feature value. Store these scores in a text file, e.g. 1BZQ_A.txt, and place the file in the Propensity_score subfolder.


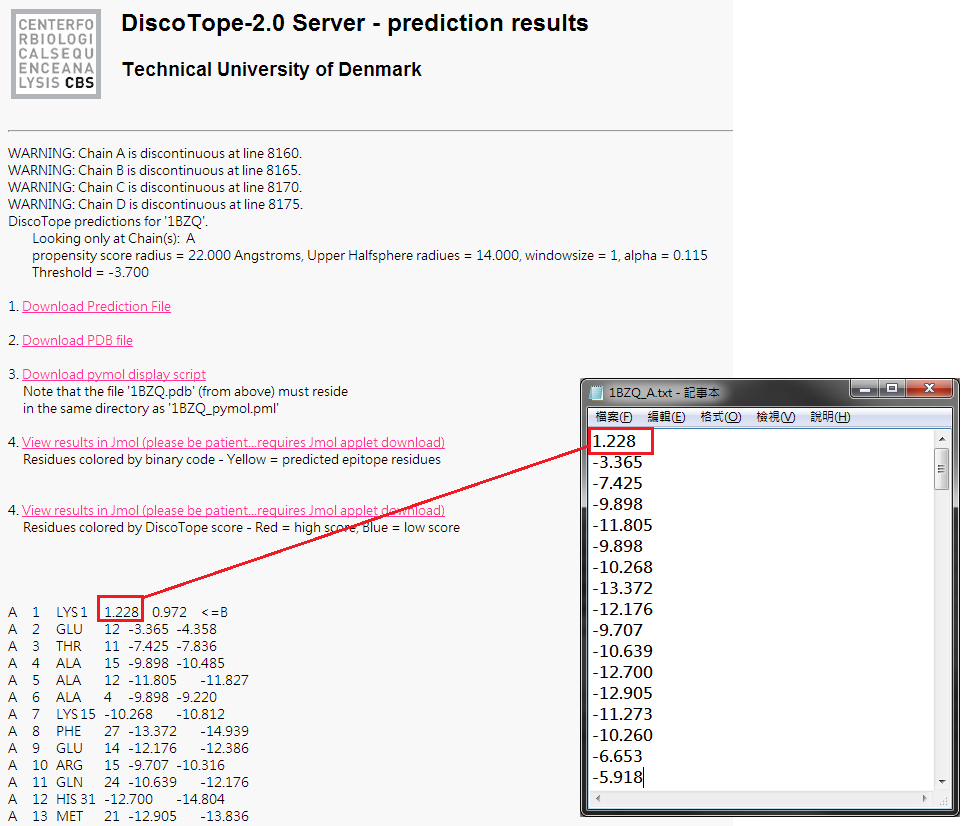


- 1. Residue accessibility

Use NACCESS (<http://www.bioinf.manchester.ac.uk/naccess/>) to calculate 4 classes of residue accessibility: total-side, main-chain, non-polar, all-polar. Store the values in text files, and place them separately in the four subfolders: Residue_accessibility_All_polar_abs, Residue_accessibility_Non_polar_abs, Residue_accessibility_Total_Side_abs, and Residue_accessibility_Main_Chain_abs.


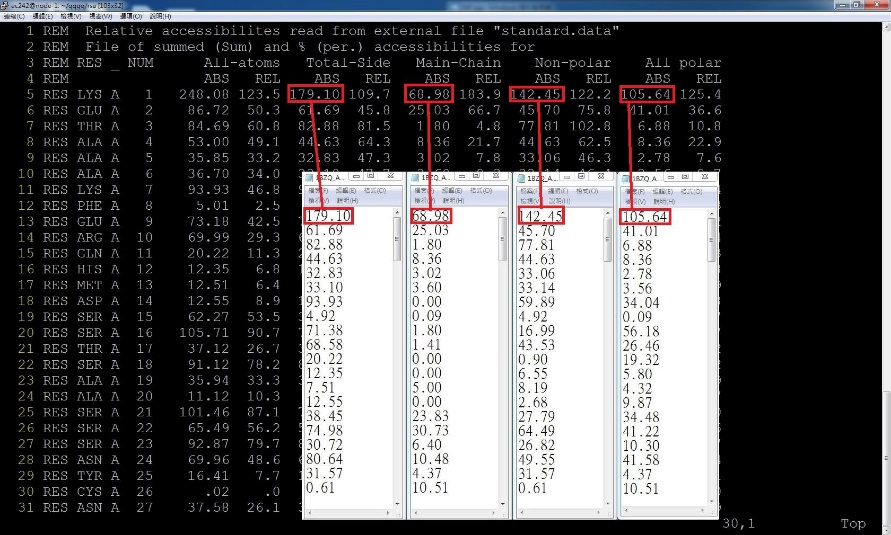


- 1. Secondary structures

Use Struc Tools (<http://helixweb.nih.gov/structbio/basic.html>) to obtain the secondary structures. They are encoded as follows.

Coil 0

Strand 1

AlphaHelix 2

Turn 3

Bridge 4

310Helix 5

PiHelix 6


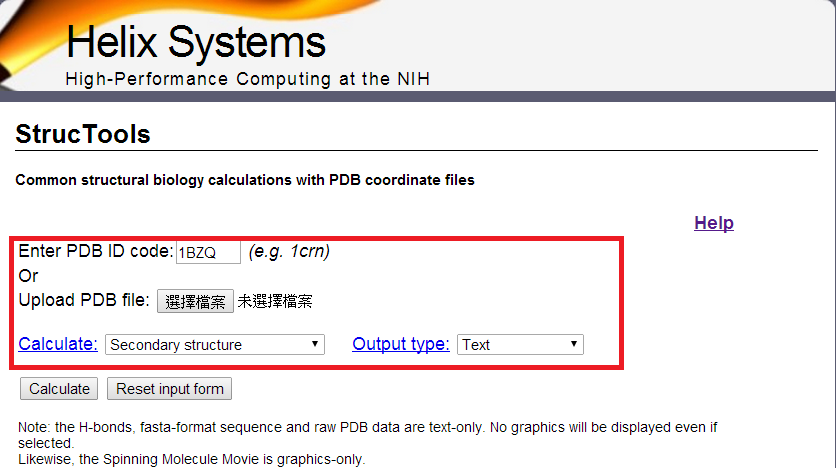


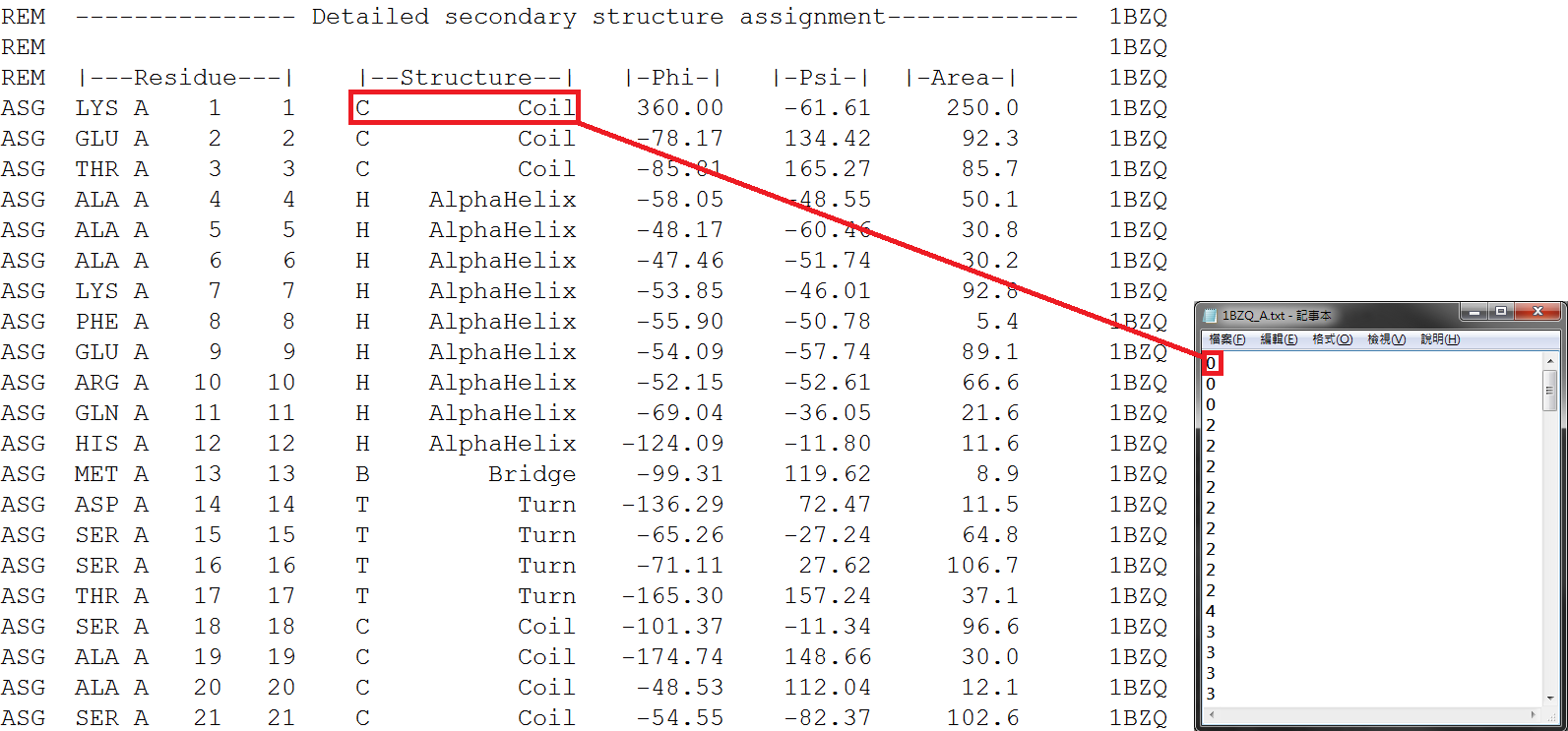


- 1. Accessible surface area

Use Struc Tools (<http://helixweb.nih.gov/structbio/basic.html>) to calculate the accessible surface area.


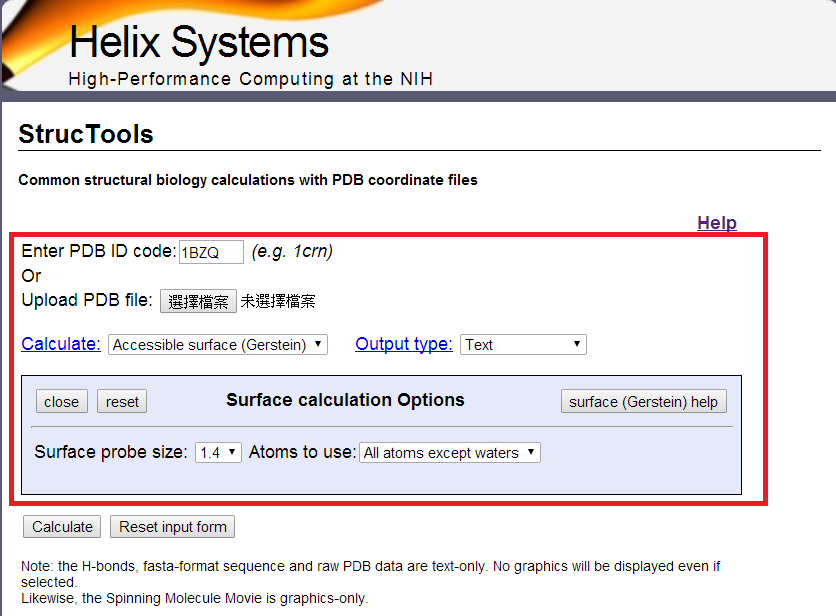


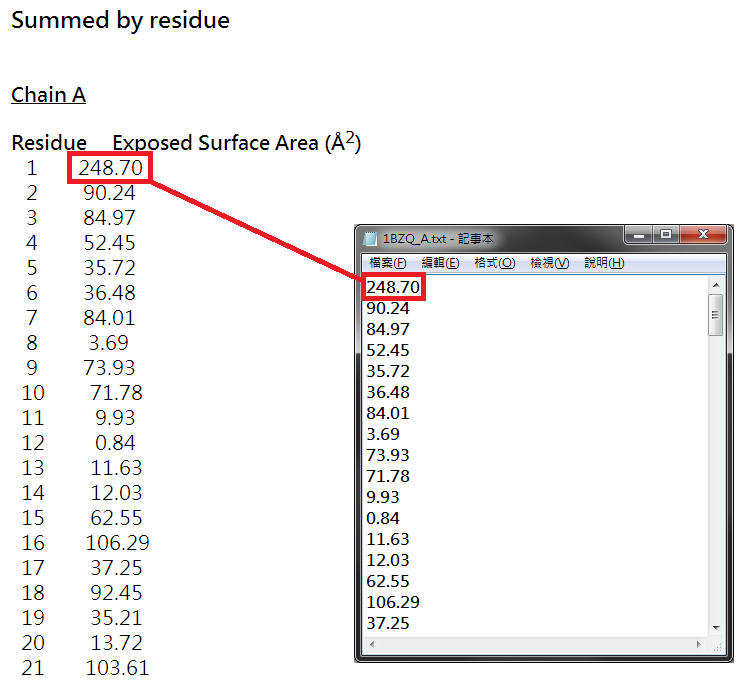


- 1. Atom volume

Use Struc Tools (<http://helixweb.nih.gov/structbio/basic.html>) to calculate the atom volume.


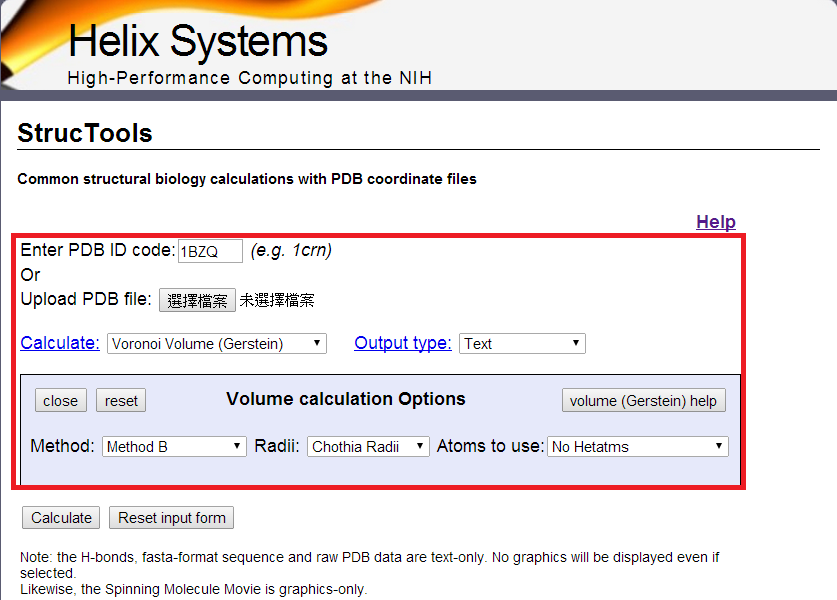


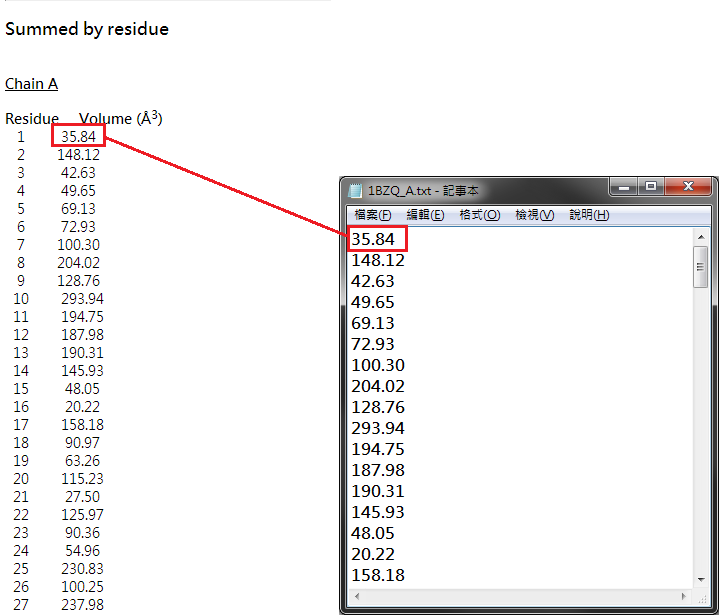


- 1. B factor

Use Struc Tools (<http://helixweb.nih.gov/structbio/basic.html>) to calculate the values of B factor.


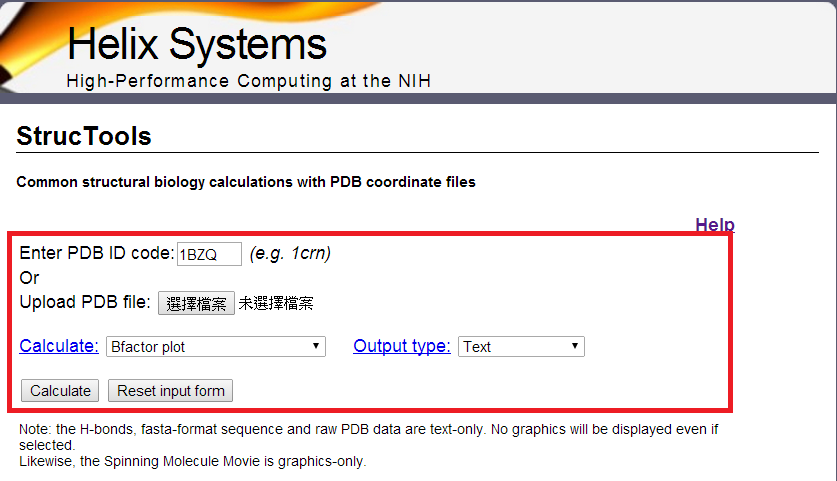


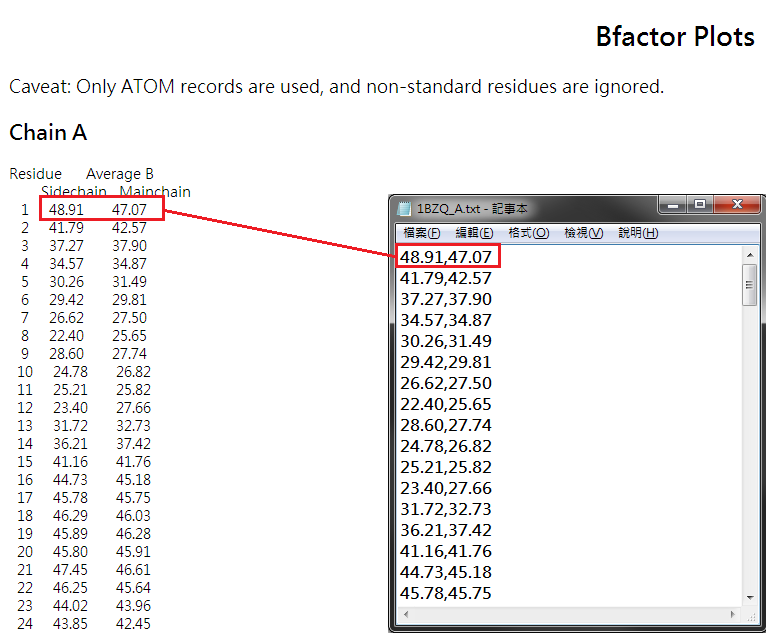


- 1. Solvent excluded surface and Solvent accessible surface

Use the MSMS program in Struc Tools (<http://helixweb.nih.gov/structbio/basic.html>) to calculate the solvent excluded surface and the solvent accessible surface.


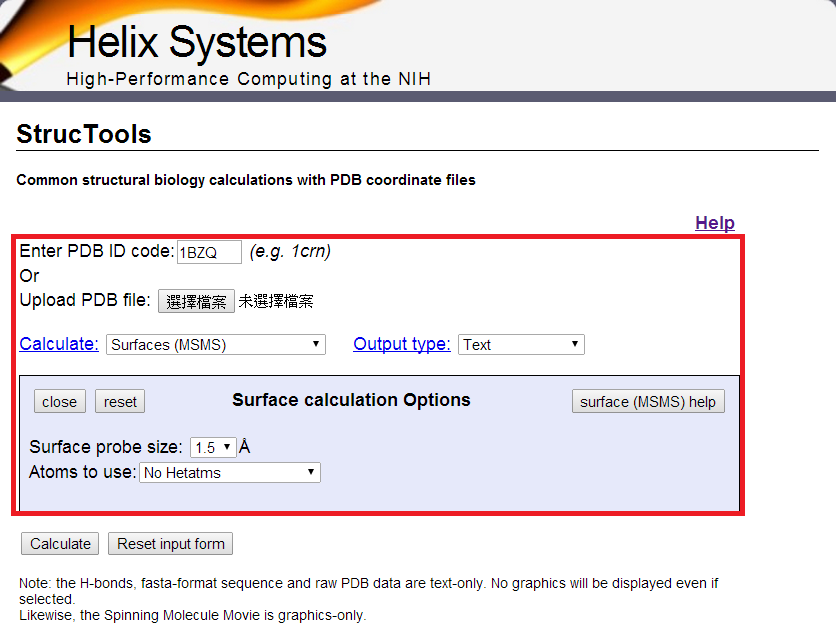


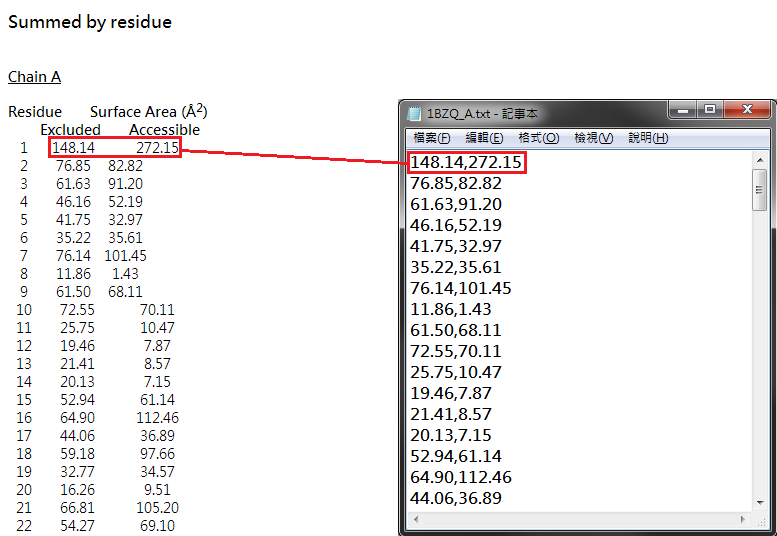


- 1. PSSM

Use BLAST (<http://blast.ncbi.nlm.nih.gov/Blast.cgi>) to obtain a PSSM, from which the information content of each position (i.e. an AA position) is derived as a base feature value.


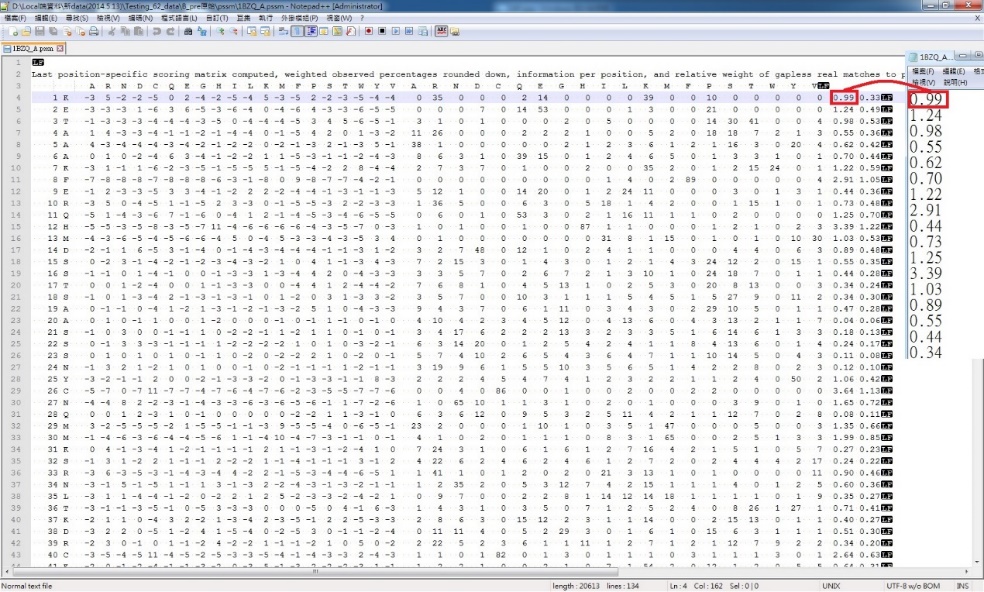


- 1. Side chain polarity and Hydropathy index

Refer to the table on <http://en.wikipedia.org/wiki/Amino_acid>.


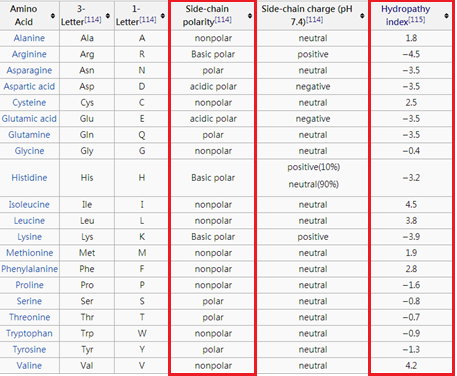


Side chain polarity is encoded as follows.

Polar 1

Basic polar 2

Acidic polar 3

Nonpolar 4


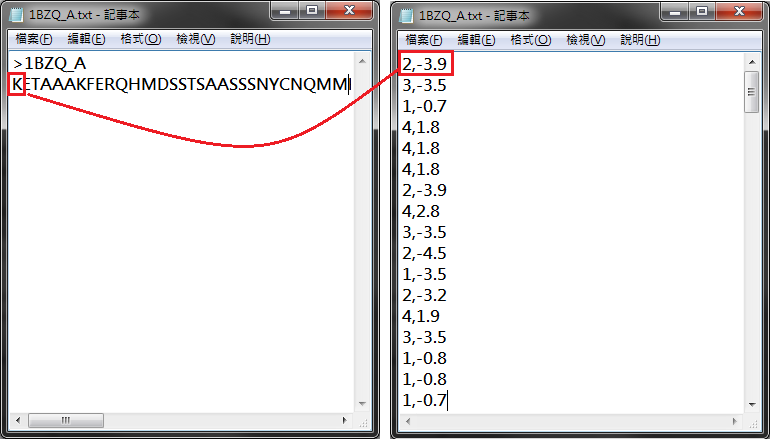


- 1. Antigenic propensity

Input protein sequences to BcePred to obtain antigenic propensity. (<http://www.imtech.res.in/raghava/bcepred/bcepred_submission.html>)


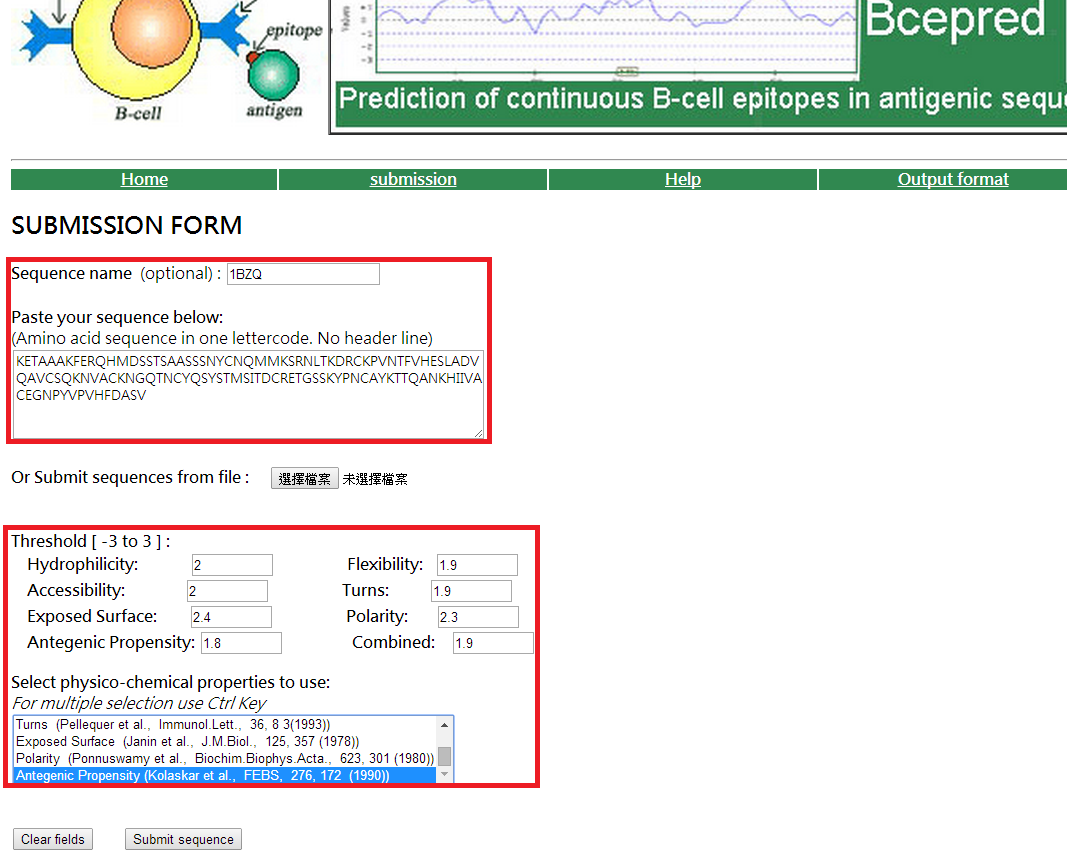


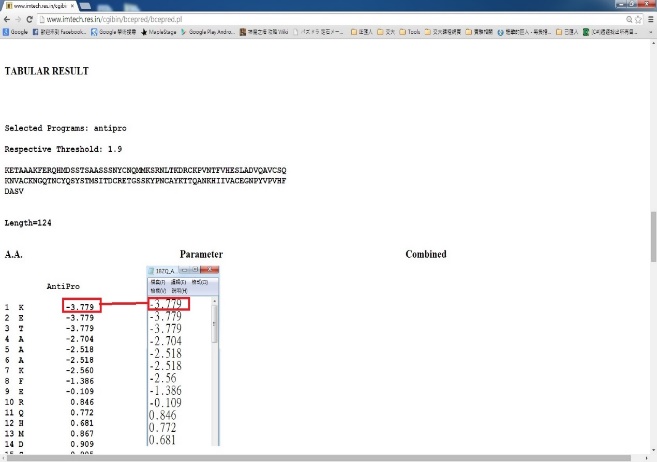


- 1. Flexibility

Input protein sequences to BcePred to obtain flexibility.

(<http://www.imtech.res.in/raghava/bcepred/bcepred_submission.html>)


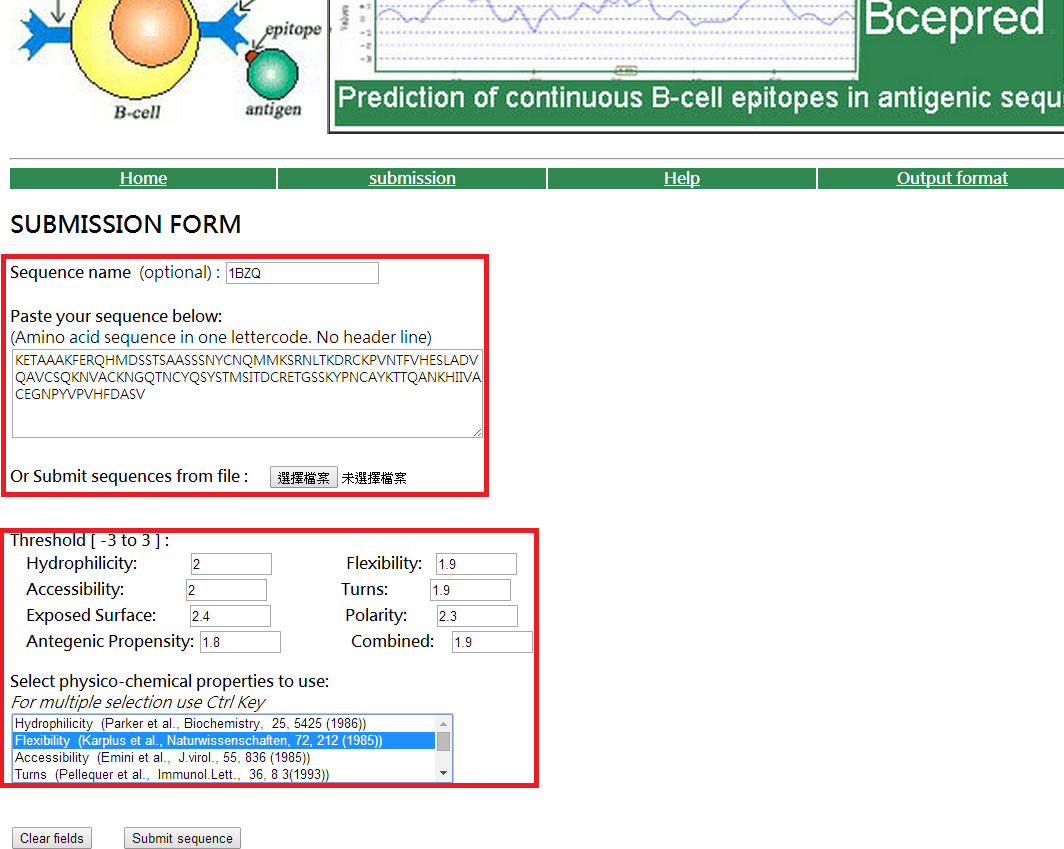


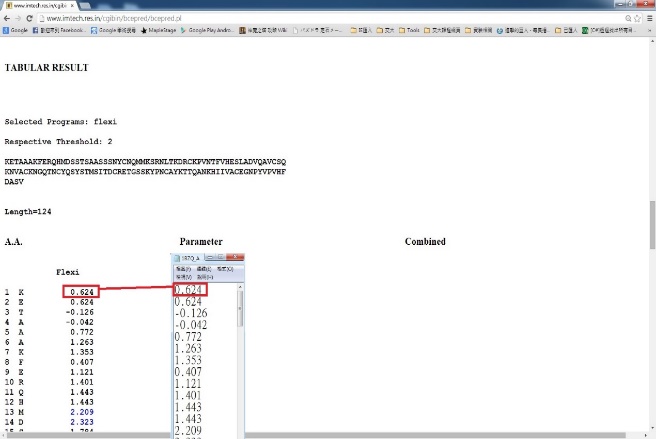


- 1. Hydrophilic scale

Input protein sequences to BcePred to obtain hydrophilic scale.

(<http://www.imtech.res.in/raghava/bcepred/bcepred_submission.html>)


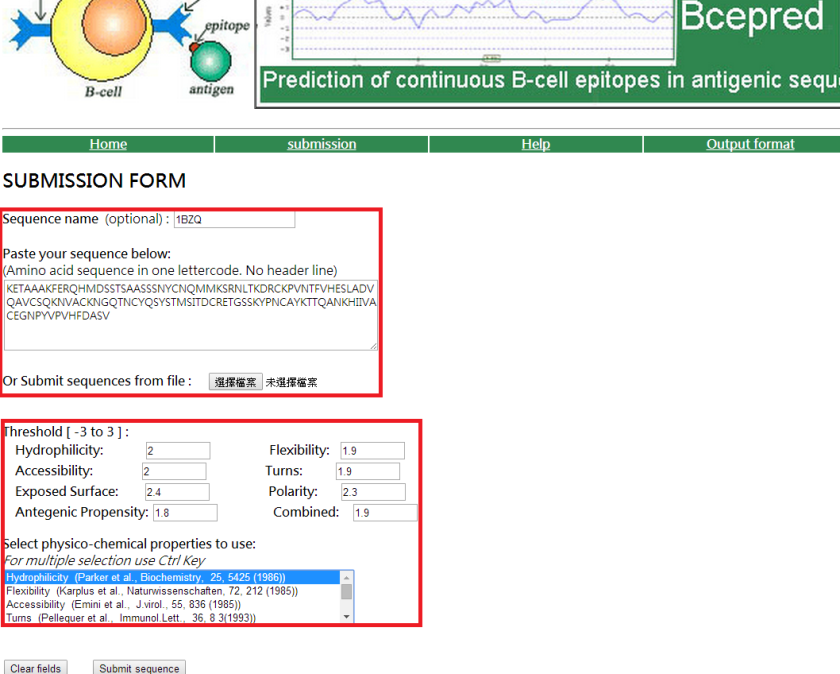


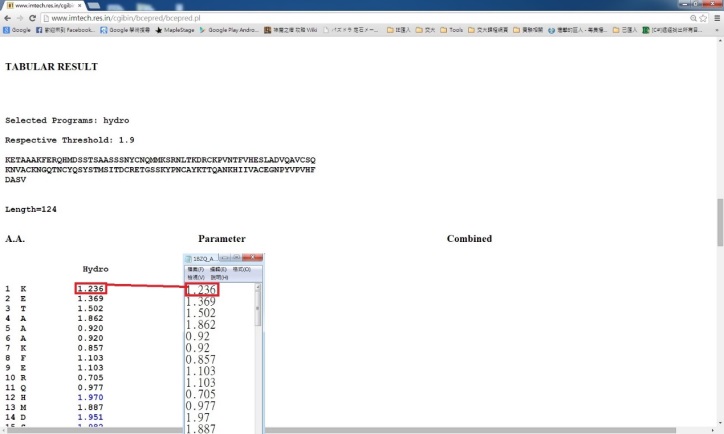

Supplement: Additional file 1: — Supplement_BMC. [file 12859_2014_378_MOESM1_ESM.docx]
